# Opposite and dynamic regulation of the interferon response in metastatic and non-metastatic breast cancer

**DOI:** 10.1186/s12964-023-01062-y

**Published:** 2023-03-07

**Authors:** Apsana Lamsal, Sonja Benedikte Andersen, Ida Johansson, Marina Vietri, Ansooya Avinash Bokil, Natalie Jayne Kurganovs, Felicia Rylander, Geir Bjørkøy, Kristine Pettersen, Miriam S. Giambelluca

**Affiliations:** 1 Department of Biomedical Laboratory Science, Faculty of Natural Sciences, Norwegian University of Science and Technology, Trondheim, Norway; 2 Centre of Molecular Inflammation Research and Department of Cancer Research and Molecular Medicine, Faculty of Medicine and Health Sciences, Norwegian University of Science and Technology, Trondheim, Norway; 3 Centre for Cancer Cell Reprogramming, Institute of Clinical Medicine, Faculty of Medicine, University of Oslo, Montebello, Oslo Norway; 4 Department of Molecular Cell Biology, Institute for Cancer Research, Oslo University Hospital, Montebello, Oslo Norway; 5 Institute for Cancer Research, Department of Tumor Biology, Oslo University Hospital, Montebello, Oslo Norway; 6 Department of Circulation and Medical Imaging, Faculty of Medicine and Health Sciences, Norwegian University of Science and Technology, Trondheim, Norway; 7 Department of Clinical Medicine, Faculty of Health Science, UiT-The Arctic University of Norway, Tromsø, Norway

**Keywords:** 4T1 model, 66cl4, 67NR, IFN-I, Metastasis

## Abstract

**Background:**

To our current understanding, solid tumors depend on suppressed local immune reactions, often elicited by the interaction between tumor cells and tumor microenvironment (TME) components. Despite an improved understanding of anti-cancer immune responses in the TME, it is still unclear how immuno-suppressive TME are formed and how some cancer cells survive and metastasize.

**Methods:**

To identify the major adaptations that cancer cells undergo during tumor development and progression, we compared the transcriptome and proteome from metastatic 66cl4 and non-metastatic 67NR cell lines in culture versus their corresponding mouse mammary primary tumors. Using confocal microscopy, RT-qPCR, flow cytometry and western blotting, we studied the signaling pathway and the mechanisms involved. In addition, we used public gene expression data from human breast cancer biopsies to evaluate the correlation between gene expression and clinical outcomes in patients.

**Results:**

We found that type I interferon (IFN-I) response was a key differentially regulated pathway between metastatic and non-metastatic cell lines and tumors. The IFN-I response was active in metastatic cancer cells in culture and markedly dampened when these cells formed primary tumors. Interestingly, the opposite was observed in non-metastatic cancer cells and tumors. Consistent with an active IFN-I response in culture, the metastatic cancer cells displayed elevated levels of cytosolic DNA from both mitochondria and ruptured micronuclei with concomitant activation of cGAS-STING signaling. Interestingly, decreased IFN-I-related gene expression in breast cancer biopsies correlated with an unfavourable prognosis in patients.

**Conclusion:**

Our findings show that IFN-I response is dampened in the tumors with the metastatic ability and lower IFN-I expression predicts poor prognosis in triple-negative and HER2 enriched breast cancer patients. This study highlights the possibility of reactivating the IFN-I response as a potential therapeutic strategy in breast cancer.

**Graphical abstract:**

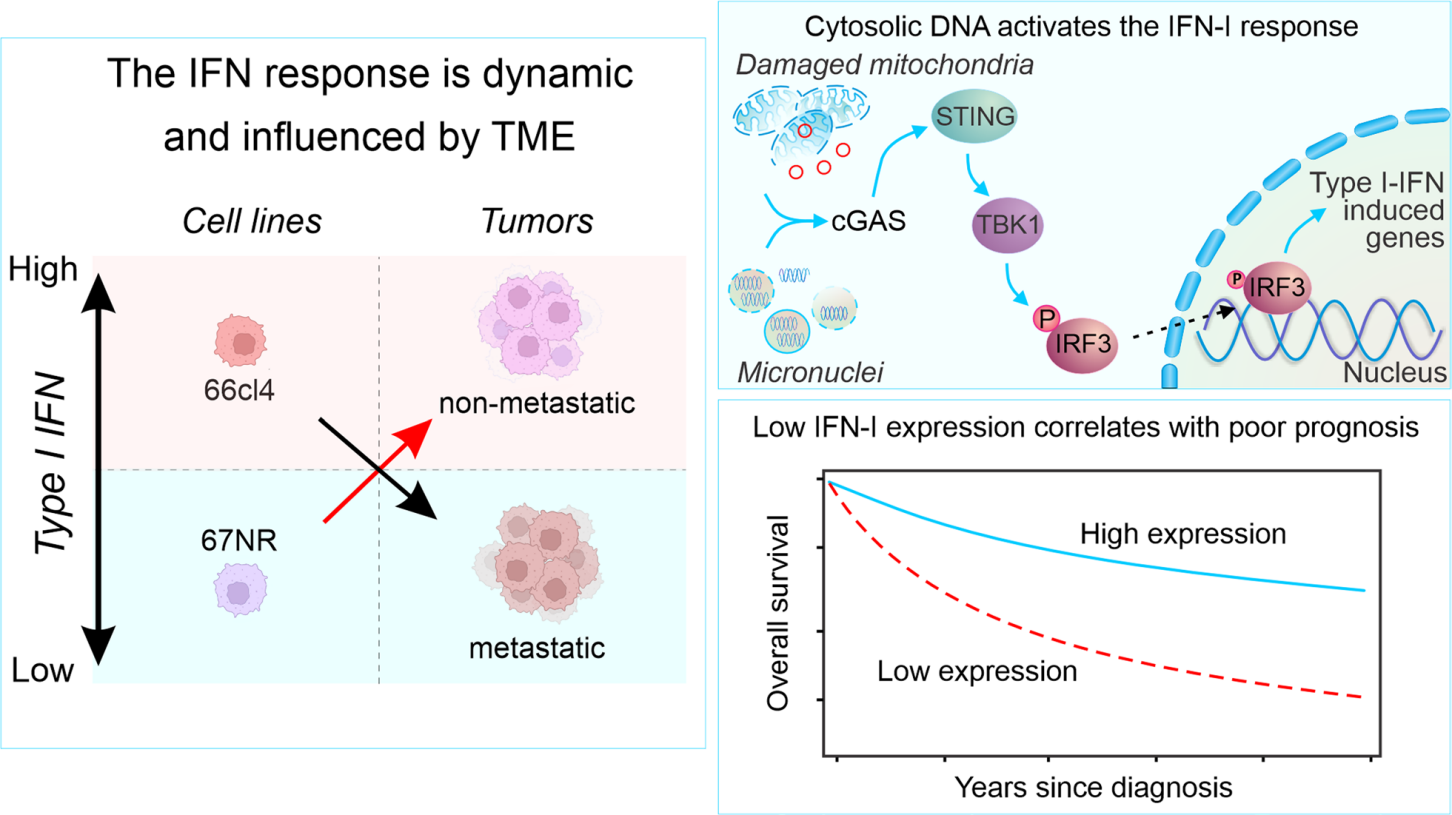

**Video Abstract**

**Supplementary Information:**

The online version contains supplementary material available at 10.1186/s12964-023-01062-y.

## Background

The interaction between cancer cells and the tumor microenvironment (TME) profoundly impacts tumor development by influencing processes that lead to either tumor eradication or tumor progression and metastasis [1–4]. In a solid tumor, the transformed cancer cells co-evolve with the TME, which includes fibroblasts, endothelial cells and infiltrating immune cells, blood vessels, signaling molecules, secreted factors, and extracellular matrix [5]. Immune cells are essential components of the TME since a proper antitumor immune response will destroy the transformed cancer cells, while a protumor immune response may support tumor growth and metastasis. Cancer cells can avoid immune recognition by actively suppressing antitumor immune responses by releasing anti-inflammatory cytokines, recruiting immunosuppressive immune cells, and shaping the TME towards a more permissive state [6–14].

Interferons (IFNs) have a crucial role in the immune response against infections, intracellular pathogens, and cancer cells. These proteins are released by infected or transformed cells and activate the immune response that promotes cytokine production, natural killer cell functions, and antigen presentation [15, 16]. Type I IFNs (IFN-I), the largest class of IFNs, have a pivotal role in cancer prevention, inducing anti-tumor immunity [17]. Downregulation of IFN-I response prevents CD8^+^T cell-mediated recognition and elimination of tumor cells. For instance, loss of the type I interferon receptor chain (IFNAR1) in colorectal cancer models led to aggressive cancer growth, while the activation of IFN-I response increases the CD8^+^T cell effector function and tumor regression [10, 17, 18]. In breast cancer models, downregulation of interferon regulatory factor (Irf7) target genes was associated with increased bone metastasis and reduced survival in this model. On the other hand, high expression of Irf7 regulatory genes correlated with increased metastasis-free survival in more than 800 patients studied [19].

IFN-I expression can be induced by activating the cGAS-STING pathway, which induced tumor regression in breast, colon cancer and melanoma mouse models when STING agonists were administered [20–24]. Moreover, STING agonists are currently used in clinical trials in combination with chemotherapy or Programmed Cell Death Ligand 1 (PDL1) antibodies highlighting the importance of IFN in cancer treatment [25]. However, a better understanding of the mechanism that controls IFN-I responses and its relationship in the TME components is needed to extend the success of this combined therapy.

Despite our improved understanding of anticancer immune responses in the TME, it is still unclear how immuno-suppressive TME are formed and how some cancer cells survive and metastasize [26]. We aimed to identify specific adaptations of metastatic cancer cells that enable them to grow in the TME, avoiding immune response and facilitating tumor progression and metastasis. We used cell lines derived from the well-established metastatic mammary carcinoma model 4T1 with different metastatic capacities. We used 66cl4 cells that metastasize to the lungs after injection into the mammary fat pad of mice, while 67NR cells do not metastasize [27]. Using the unbiased approach of transcriptomics and proteomics to compare metastatic and non-metastatic cancer cells grown in culture versus their corresponding tumors, we showed that the IFN-I response differed. Specifically, we found a significant dampening in the IFN-I response in the metastatic tumors compared to the cells in the culture. In contrast, an increase in the IFN response was observed in the non-metastatic tumor. In addition, we found that elevated IFN-I response in metastatic cancer cells was related to the high cytosolic DNA levels and activation of their sensor system. Our results suggest that factors in the TME enable metastatic tumors to silence their IFN-I response, thus avoiding the antitumor immune response. Hence, a better understanding of the mechanism used by metastatic tumors to dampen the local IFN-I signaling could lead to novel targeted therapies to reactivate local immune reactions and boost responses to conventional therapies.

## Methods

### Cell lines and cell culture

67NR and 66cl4 cells, obtained from Barbara Ann Karmanos Cancer Institute, and MDAMB453 and MDAMB231 cells, kindly provided by Dr. Kaisa Lehti were cultivated as described in Additional file 1: Methods.

### Transcriptome analysis

RNA from 66cl4 and 67NR cells, as well as from 66cl4 and 67NRprimary mammary tumors from BALB/cJ mice were isolated and sequenced as described before [28] and analyzed as in Additional file 1: Methods.

### Mice experiments

Eight- to twelve-week-old female BALB/cJ mice were obtained from Janvier Labs, France. The tumors were initiated and resected and processed as in Additional file 1: Methods.

### Mass spectrometry analysis

Proteins were isolated from 66cl4 and 67NR mammary breast tumors from mice by homogenization in lysis buffer and analyzed by LC–MS/MS as described in Additional file 1: Methods. The proteomics data have been deposited to the ProteomeXchange Consortium via the PRIDE [29] partner repository with the dataset identifier PXD037288.

### Quantitative real-time PCR

Total RNA from cells in culture was extracted using RNeasy Mini Kit (Qiagen). RNA concentration and purity were measured by Nanodrop. cDNA synthesis followed by qPCR was performed as described in Additional file 1: Methods.

### Immunoblotting

Cells were harvested in 8 M urea lysis buffer (8 M urea, 0.5% (v/v) Triton X-100, 100 mM DTT, 1 × Complete® protease inhibitor (Roche) and 2 × phosphatase inhibitor cocktail II and III (Sigma)). When indicated, the cells were pretreated with the cGAS inhibitor (Invivogen, # inh-ru521) or the TBKI inhibitors MRT67307 and BX795 (Sigma, #HY-13018 and # HY-10514). Frozen tumor tissues were thawed in urea lysis buffer and homogenized as described under sample preparation and MS analyses in Supplementary Methods. Protein concentration was measured and subjected to western blot as in Supplementary Methods.

### ELISA

67NR and 66cl4 cells were cultured in full growth medium for three days until they reached 80–90% confluency. Conditioned medium (CM) was collected, centrifuged, and filtered through a 0.22 µm filter. CXCL10 levels were determined using Mouse CXCL10/IP-10/CRG-2 DuoSet ELISA (R&D systems, #DY466) according to the manufacturer’s protocols. The data was analyzed using Microplate Manager 6 (Bio-Rad).

### Immunofluorescence

Cells were grown on high precision cover glass until desired confluency, fixed, permeabilized, stained with antibodies specific for cGAS and Lamin A and imaged as specified in Additional file 1: Methods.

Analysis of mitochondrial membrane potential and reactive oxygen species.

Mitochondrial membrane potential (MMP) and production of reactive oxygen species (ROS) were assessed as described in Additional file 1: Methods.

### Detection of total and cytosolic mtDNA

Total and cytosolic DNA were isolated from 66cl4 and 67NR cells and subjected to qPCR as described in Additional file 1: Methods.

### Use of public databases

Kaplan–Meier plotter [30], Broad Institute Cancer Cell Line Encyclopedia (CCLE), (https://portals.broadinstitute.org/ccle) [31] and cBioPortal [32] were used as described in Additional file 1: Methods.

### Statistics

Statistical analyses were performed in GraphPad Prism 9. Values are expressed as mean ± standard deviation (SD) or standard error of the mean (SEM) if not otherwise stated. Details about statistical analyses are specified in the figure legends. *p*-value < 0.05 was considered statistically significant and is labeled with *, *p* < 0.01 is labeled with **, *p* < 0.001 is labeled with *** and *p* < 0.0001 is labeled with ****.

## Results

### IFN-I-associated gene expression is suppressed in metastatic tumors

**Fig. 1:**
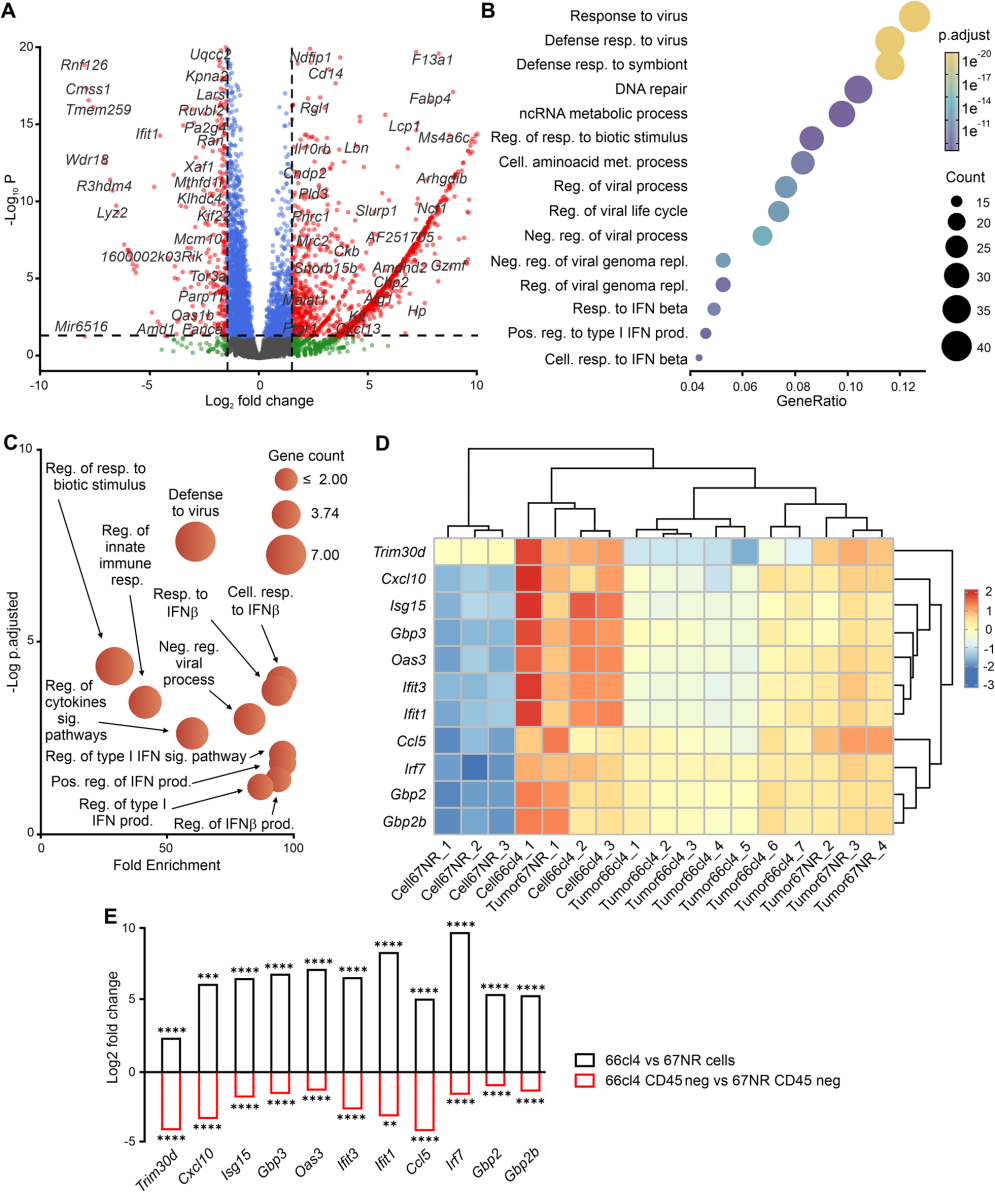
IFN-I-related gene expression is suppressed in metastatic cancer cells. **A** Volcano plots depicting differentially expressed genes from 66cl4 primary tumors vs 66cl4 cells. Red points represent genes with Log2foldchange within the cut-off (± 1.5) and adjusted *p*-value < 0.05. **B** Gene Ontology (GO) analysis of the most enriched biological processes (BP) associated with the genes with a reduced expression in 66cl4 tumor versus 66cl4 cell line. **C** Gene Ontology analysis of the most enriched biological processes of the 11 genes highly expressed in 66cl4 versus 67NR cells but low expressed in 66cl4 versus 67NR tumors. **D** Heatmap showing transcript per million (after log10 transformation) of the 11 genes that are oppositely expressed in cells in culture and in primary tumors of 67NR and 66cl4. **E** Significantly upregulated IFN-I genes in 66cl4 cells vs. 67NR cells (N = 3) and downregulated genes in 66cl4 (CD45-) vs 67NR (CD45-) population (N = 5) sorted from the primary tumors of 66cl4 and 67NR

To identify transcriptome dynamics that occur during metastatic tumor development, we compared the RNA-sequencing profile of cell cultures and tumors formed by the metastatic 66cl4 cells. This analysis identified 1859 genes that were differentially expressed between 66cl4 cells grown in culture versus their corresponding primary tumors (log2 Fold Change (FC) >  ± 1.5; adjusted *p*-value < 0.05). Of these, 1537 genes were significantly higher expressed, whilst 322 genes were significantly lower expressed in the 66cl4 tumors (Fig. 1A). To understand the biological processes (BP) linked to the differentially expressed genes, we performed gene ontology (GO) enrichment analysis. This showed that the highly expressed genes in the 66cl4 tumors were associated with inflammation and cell chemotaxis (Additional file 1: Fig.S1A) and that the lower expressed genes were associated with viral defense and IFN-I response (Fig. 1B).

To investigate if we could extend these observations to a non-metastatic tumor, we compared the transcriptomes of the non-metastatic 67NR cell line grown in culture versus its primary tumor. This analysis identified 1084 genes differentially expressed between the 67NR samples (log2FC > 1.5; adjusted *p*-value < 0.05). Of these, 938 genes were significantly elevated, whilst 146 were reduced in the tumors (Additional file 1: FigS1B). GO analysis for BP revealed that the high-expressed genes were also involved in inflammation and leukocyte migration (Additional file 1: Fig.S1C), while the low-expressed genes were related to RNA metabolism (Additional file 1: Fig.S1D). Together, the results obtained from the 66cl4 and 67NR analyses showed that genes with a lower expression are involved with different BP in the metastatic and non-metastatic tumors, raising the possibility that these signaling pathways are associated with the different metastatic ability of the tumors.

To further understand the dynamic changes in gene expression between cell lines and tumors, we compared the differentially expressed genes in 66cl4 and 67NR, both when grown in culture and when forming primary tumors (Additional file 1: Fig.S2). Compared to the 67NR cells, 411 genes were highly expressed in 66cl4 cells (log2FC > 1.5). Strikingly, 11 genes stood out as being significantly elevated in 66cl4 cells in culture but also among the significantly downregulated genes in the 66cl4 tumors. GO analysis for BP of these 11 genes revealed that they were involved in IFN response, especially in IFN-I signaling (Fig. 1C). Interestingly, these 11 transcripts were amongst those showing low expression in 67NR cells in culture but higher expression in the 67NR tumors (Fig. 1D).

To confirm that the expression of these 16 genes was reduced in metastatic cancer cells following tumor formation, we performed RNA sequencing from isolated non-immune cell-enriched (CD45-negative) and immune cell-enriched (CD45-positive) from 66cl4 and 67NR tumors. This analysis showed a significantly lower expression of these 11 genes in 66cl4 isolated from CD45-negative population enriched with cancer cells compared to (CD45-negative) population from 67NR (Fig. 1E, Additional file 1: Fig.S3A). The reduction in expression of 11 genes was also observed in the CD45-positive population from 66cl4 tumor (Additional file 1: Fig S3B). This suggests that metastatic and non-metastatic cells utilize different strategies to successfully form a tumor, and that alterations in IFN-I signaling stand out as strikingly different.

### Type I IFN-associated proteins are lower in the metastatic tumor

**Fig. 2:**
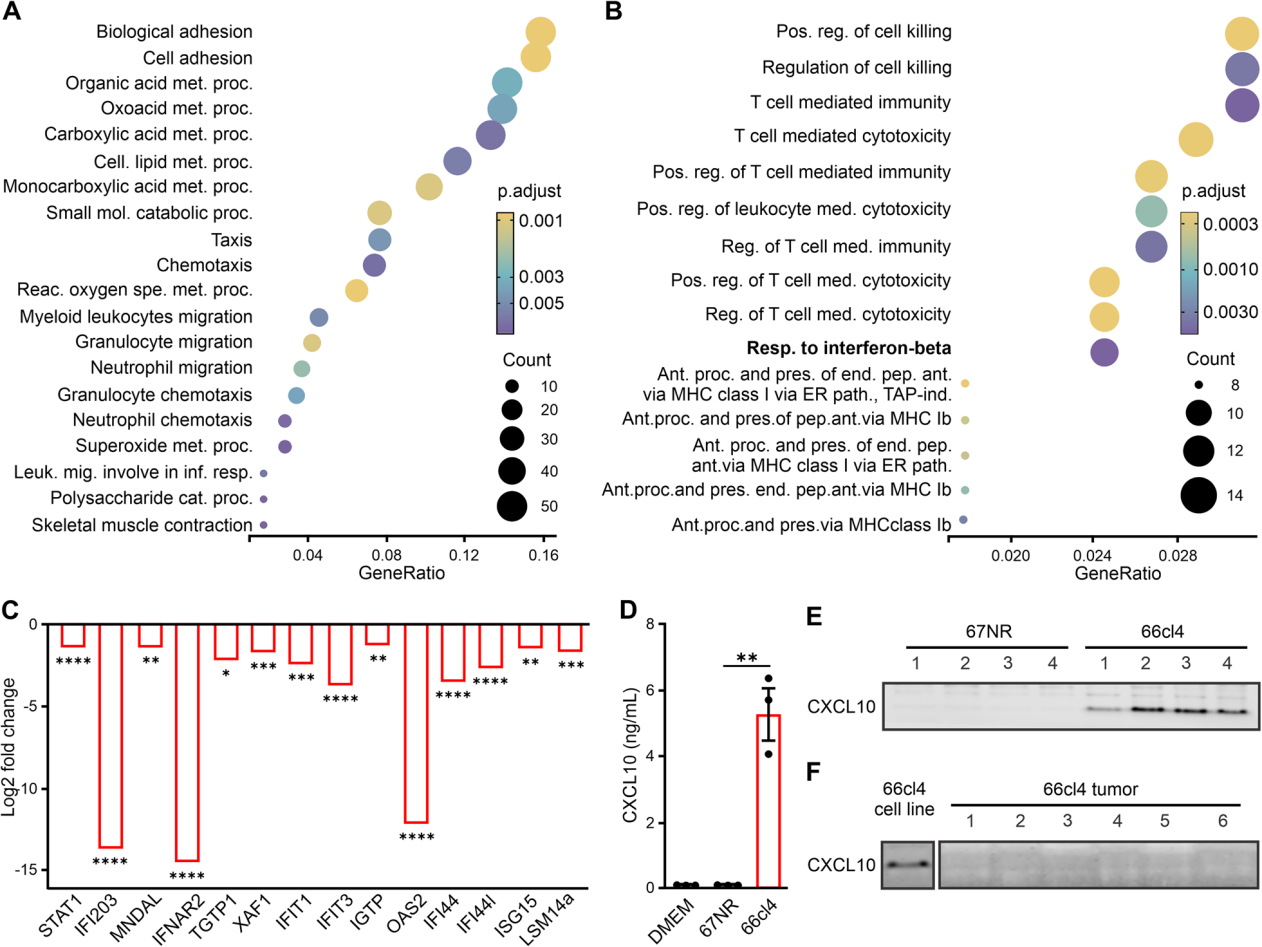
IFN-I proteins are dampened in metastatic tumors. **A** GO (BP) functional enrichment analyses of proteins with elevated levels in 66cl4 tumors (N = 6) relative to 67NR tumors (N = 5), from MS analysis (log2FC ≥ 1.5, *p*-value < 0.05). **B** GO (BP) functional enrichment analyses of proteins with a reduced expression in 66cl4 tumors (N = 6) relative to 67NR tumors. **C** Significantly downregulated IFN-I proteins in 66cl4 tumors. **D** CXCL10 levels in conditioned medium from 67NR and 66cl4 cells determined by ELISA. Bars represent means ± SEM (N = 3). E) CXCL10 immunoblot of protein extracts from 67NR and 66cl4 cell lines (N = 4). F) CXCL10 immunoblot of protein extracts from 66cl4 cell line (N = 1) and 66cl4 tumors (N = 6). Statistical significance was determined using Student’s t-test (*p < 0.05, **p < 0.01, ***P < 0.001 and ****p < 0.0001)

To investigate whether the transcriptional differences associated with the IFN-I response in metastatic and non-metastatic tumors correlate with protein levels, we analyzed the proteomes of the 66cl4 (n = 6) and 67NR (n = 5) primary tumors. Principal component analysis of the 5906 detected proteins showed a high degree of similarity between the biological replicates of each tumor (Additional file 1: Fig. S4A). In addition, the tumor samples were separated by tumor type (67NR vs 66cl4) based on the relative abundance of individual proteins, indicating differential protein levels between them (Additional file 1: Fig.S4A). Compared with 67NR tumors, 66cl4 tumors displayed elevated levels of 387 proteins (log2FC > 1.5) and lower levels of 328 proteins (log2FC < − 1.5) (Additional file 1: Fig. S3B). To identify the main biological processes associated with the identified proteins, we performed GO (BP) enrichment analyses of the tumor proteomes. Consistent with the RNA sequencing-based data, primary tumors formed by 66cl4 expressed higher levels of proteins related to leukocyte migration and chemotaxis (Fig. 2A). In contrast, proteins related to adaptive immune response and cytotoxicity were markedly reduced in 66cl4 tumors versus 67NR tumors (Fig. 2B). We also noticed significantly lower levels of interferon beta-associated proteins in metastatic 66cl4 tumors versus 67NR tumors (Fig. 2B), suggesting that reduced transcription of the interferon-related genes in metastatic tumors correlates with a significant decrease in IFN-I associated proteins.

We further examined the proteins associated with the response to interferon beta and other interferon-related proteins selected based on the literature [33]. We found low levels of 14 IFN-related proteins among the less abundant proteins (log2FC <  − 1.5) in 66cl4 tumors (Fig. 2C). These were compared with the 11 ‘oppositely’ expressed genes that were identified earlier (high in 66cl4 vs. 67NR cells in culture but low in 66cl4 vs 67NR in tumors), and six proteins were detected by proteomics. Amongst these, three proteins corresponded to transcripts that were dampened in 66cl4 tumors (Fig. 1D): IFIT3, IFIT1 and ISG15. Two proteins, OAS3 and GBP2, had significant lower expression in 66cl4 tumors versus 67NR tumors, however, these were outside the cutoff of log2FC < − 1.5. Only one protein, GBP2B, did not correlate with its detected mRNA levels. Among the five undetected proteins in MS (CXCL10, TRIM30d, IRF7, GBP3 and CCL5), we chose CXCL10, CCL5 and IRF7 for further validation [34, 35]. This analysis showed that both mRNA and protein levels of CXCL10, CCl5 and IRF7 protein levels were elevated in metastatic cancer cells in culture and reduced in 66cl4-derived primary tumors (Fig. 2D–F and Additional file 1: Fig.S4C–I). Together, the transcriptomic and proteomic analyses of the cancer cells in culture and primary tumors indicate the dampening of the IFN-I response as the most evident adaptation during tumor formation in the metastatic model.

### Cytosolic nuclear DNA is elevated in the murine metastatic and aggressive human cancer cells

**Fig. 3:**
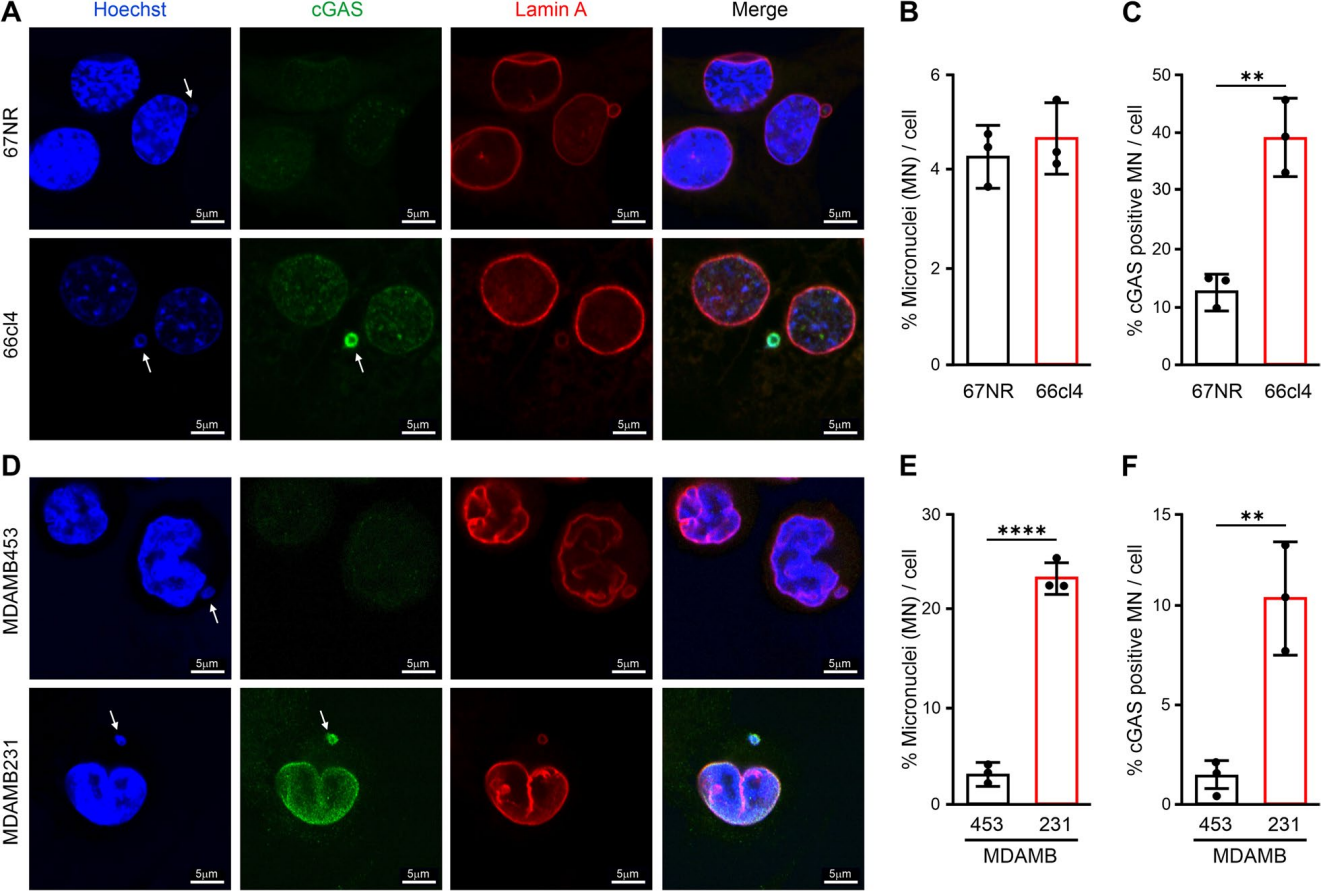
Metastatic and invasive cancer cells display elevated levels of cGAS-positive micronuclei. **A** Representative immunofluorescence staining of micronuclei in 67NR and 66cl4 cells with cGAS (Green) and Lamin A (red) antibodies. Micronuclei and cGAS-positive micronuclei are highlighted by white arrows. DNA was stained with Hoechst (Blue). Scale bar: 5 μm. **B**–**C** Percentage of micronuclei per cell (**B**) and cGAS-positive micronuclei per cell (**C**) calculated from three independent experiments (N =  > 1000 cells per experiment, from three independent experiments). **D**–**F** Representative immunofluorescence staining and quantitation of micronuclei and cGAS-positive micronuclei in MDAMB453 and MDAMB231 cells (N =  > 1000 cells per experiment, from three independent experiments). Scale bar: 5 μm. Bars represent mean ± SEM (**p < 0.01, ****p < 0.0001; Student’s t-test)

The data presented above is consistent with a constitutive IFN-I response in metastatic 66cl4 cells in culture that is downregulated in tumors formed by these cells. Thus, the response is dynamically regulated. We next wondered how the IFN-I response can be constitutively activated in sterile cell culture conditions. IFN-I response is induced by cytosolic DNA [36]. Often cancer cells contain cytosolic micronuclei produced by chromosomal mis-segregation events during mitosis [37]. Immunostaining of DNA and confocal microscopy revealed a similar number of micronuclei in the metastatic and non-metastatic cells (Fig. 3A, B). However, the activation of the IFN-I signaling depends on cGAS physically binding to DNA, a condition that is met at a subset of micronuclei characterized by a ruptured nuclear envelope [37, 38]. Strikingly, the fraction of cGAS-positive micronuclei was considerably higher in 66cl4 cells than in 67NR (Fig. 3C). Together, transcriptome, proteome and signaling analyses suggest that cGAS binds to DNA in the cytosol of 66cl4 cells in culture and cause a constitutive IFN-I response in these metastatic cancer cells that is not detected in the non-metastatic cells. As 66cl4 and 67NR are mouse-derived breast cancer cell lines, we next postulated whether similar differences in IFN-I expression could be identified in human breast cancer cell lines. We then analyzed the expression of two well-known interferon-induced genes, IFIT3 and IFI44, using mRNA expression data from the Cancer Cell Line Encyclopedia. Interestingly, the expression of these two transcripts showed a strong association with each other (Additional file 1: Fig.S5A). Still, their expression varied among the different cancer cells, including the 60 breast cancer cell lines in the database (Additional file 1: Fig. S5B). For instance, IFIT3 and IFI44 mRNA were highly expressed in the invasive human breast cancer cell line MDAMB231 [39, 40], while their expression was low in the non-invasive MDAMB453 (Additional file 1: Fig. S5B). These findings were confirmed by transcript and protein quantification of IFIT3 in cell extracts of MDAMB231 and MDAMB453 cells (Additional file 1: Fig. S5C–E). In line with the observations seen in mouse breast cancer cell lines, a higher number of micronuclei and cGAS-positive micronuclei were observed in the cytosol of the invasive MDAMB231 compared to non-invasive MDAMB453 human cancer cells (Fig. 3D–F). Together, our data suggest that the IFN-I response could be associated with constitutive activation of the DNA sensor cGAS by recognising nuclear DNA in the cytosol of metastatic and aggressive cancer cells.

### Mitochondrial DNA in the cytosol triggers IFN-I response in metastatic cancer cells

**Fig. 4:**
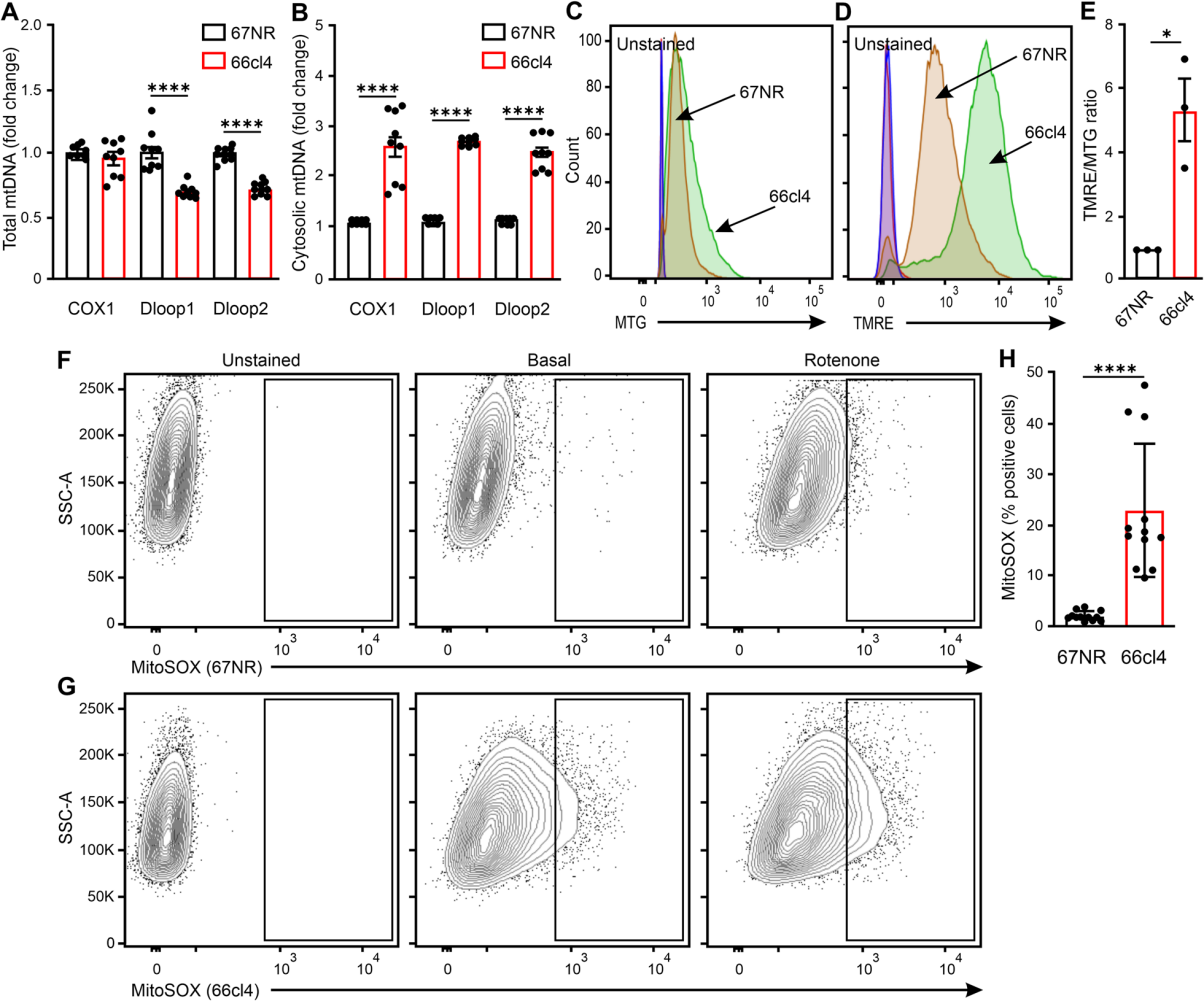
Mitochondrial DNA release into the cytosol in metastatic cell lines associates with mitochondrial stress. **A** Relative amount of total (**A**) and cytosolic (**B**) mitochondrial DNA (mtDNA) in 67NR and 66cl4 cells, normalized to 18s. Bars represent mean ± SEM (N = 3, each in triplicate, one sample t-test, ****p < 0. 0001). **C**–**D** Representative histograms of MTG (**C**) and TMRE (**D**) in 67NR and 66cl4 cells. **E** TMRE/MTG ratio in 67NR and 66cl4 cells. Bars represented mean ± SEM (N = 3, one sample t-test, *p < 0.05). **F**–**G** Representative histograms showing MitoSox positive populations in 67NR (**F**) and 66cl4 cells (**G**). Rotenone was used as a positive control. Black box represents the gating strategy to identify positive cells. **H** Bars represent mean ± SEM of MitoSOX positive cells in 67NR and 66cl4 cells (N = 4, t-test, and ****p < 0. 0001)

The IFN-I response can be activated by cytosolic DNA from other sources, such as mitochondria. We therefore measured the levels of mitochondrial DNA (mtDNA) from intact cells and the cytosolic fractions of 66cl4 and 67NR cells. The mtDNA was measured via qPCR using different mtDNA primers (COX1, Dloop1 and Dloop2). Total mtDNA levels were lower in the 66cl4 cells than 67NR (Fig. 4A and Additional file 1: Fig.S6A). However, in the cytosolic fraction, significantly higher levels of mtDNA were detected in 66cl4 cells (Fig. 4B and Additional file 1: Fig.S6B).

Mitochondrial stress can trigger the release of mtDNA into the cytosol. We, therefore, asked if mitochondria function differently between the metastatic and non-metastatic cells. We performed flow cytometry analysis of the mitochondria mass (mitotracker green; MTG), membrane potential (tetramethylrhodamine ethyl ester perchlorate; TMRE) and mitochondrial ROS production (MitoSox) using fluorescent probes. Despite the mitochondrial mass being similar between the cancer cells, the membrane potential was higher in 66cl4 compared with 67NR cells (Fig. 4C–D). Indeed, the TMRE/MTG ratio, was significantly higher in 66cl4 cells, indicating hyperpolarized mitochondria (Fig. 4E). To further understand if hyperpolarized mitochondria is associated with mtROS production in 66cl4 cells, we quantified mtROS levels. This showed a higher number of 66cl4 cells were positive for mtROS compared to 67NR cells at the basal state (Fig. 4F–H). Furthermore, when the cells were treated with an electron transport chain inhibitor (rotenone), 67NR cells showed a six-fold increase in mtROS levels, while 66cl4 cells showed less than a two-fold increase (Additional file 1: FigS6C). These results suggest that mitochondria in the metastatic 66cl4 cells work at maximum capacity.

Altogether, these data indicate that metastatic cells have poor mitochondrial quality leading to mitochondrial stress and mtDNA release in the cytosol.

### The cGAS-STING pathway regulates IFN-I response in metastatic cancer cells

**Fig. 5:**
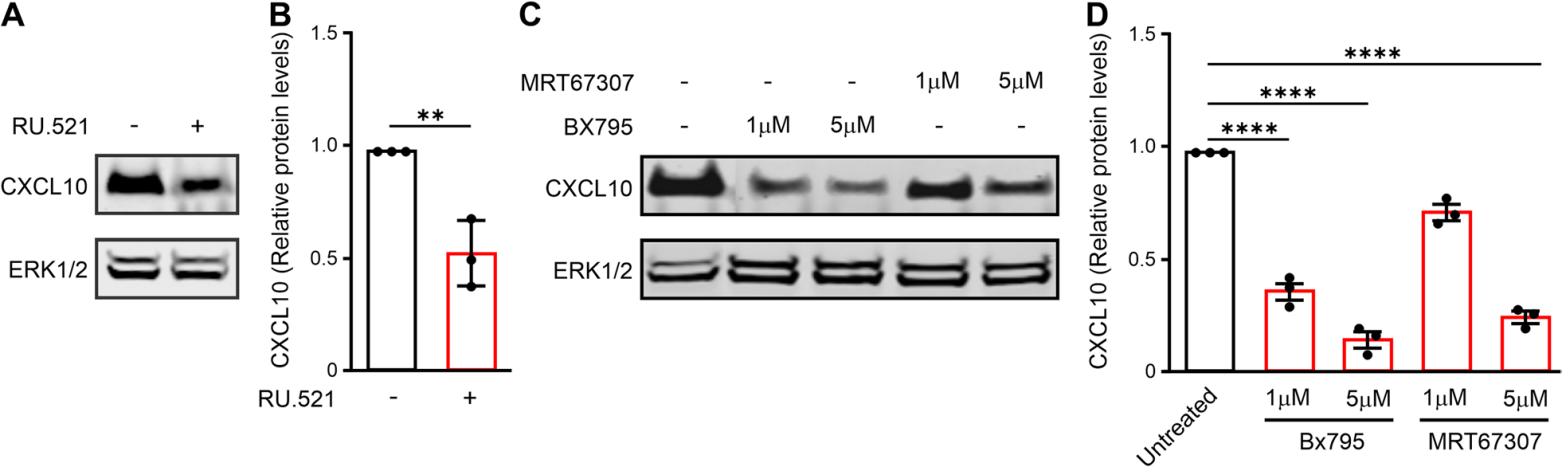
IFN-I expression in invasive cancer cells is dependent on cGAS-TBK1 signaling. **A** Representative CXCL10 and ERK1/2 immunoblot of protein extracts of 66cl4 cell line treated with and without cGAS inhibitor (RU.521, 6 µM, 24 h). **B** Quantification of A. Bars represented mean ± SEM relative to ERK1/2 (N = 3, one sample t-test, **p < 0.01). **C** Representative CXCL10 immunoblot of protein extracts of 66cl4 cell lines treated with and without TBK1 inhibitors (BX795: 1 µM, 5 µM and MRT67307: 1 µM, 5 µM, 6 h). **D** Quantification of C. Bars represented mean ± SEM relative to ERK1/2 (N = 3, ANOVA, Dunnett’s multiple comparisons test, ****p < 0.0001)

Our results show that the IFN-I response is constitutively active in the metastatic cancer cells in culture, while the IFN-I response is dampened when these metastatic cancer cells form a tumor. A better understanding of the mechanisms that activate this response is important to comprehend how this response could be reactivated in tumors as a therapeutic strategy. Cytosolic DNA is sensed by the cGAS-STING pathway, which is triggered in response to foreign or self-DNA and can activate the IFN response [41]. Transcriptome analysis [28] and qPCR validation showed that the metastatic 66cl4 cells had higher *Sting *mRNA than 67NR cells (Additional file 1: Fig.S7A). In line with this, STING protein level was also higher in 66cl4 (Additional file 1: Fig.S7B). Upon activation, STING recruits and phosphorylates TBK1 and IRF3 to induce the production of type I IFNs [42]. Chemical inhibition of cGAS or TBK1 led to significantly reduced production of the IRF3 target CXCL10 in 66cl4 cells (Fig. 5A–D), indicating that the cGAS-STING-TBK1 pathway is important for IFN-I expression in the metastatic mouse breast cancer cells. Also, in invasive human breast cancer cells MDAMB231, the protein level of STING and phosphorylated TBK1 was higher than in the non-invasive MDAMB453 cells (Additional file 1: Fig.S7C-D). Together, these results indicate that elevated IFN-I response in both mouse and human breast cancer cell cultures is associated with activation of the cGAS-STING pathway. The markedly dampened IFN-I in tumors formed from these invasive/metastatic cell lines suggest that the signaling in this pathway is disrupted in vivo to allow such cancer cells to form a metastatic tumor.

### Lower IFN-I expression correlates with a poor prognosis in breast cancer patients

To examine if an active IFN-I response has a prognostic relevance in breast cancer patients, we performed a meta-analysis using the Kaplan–Meier plotter database [30] of gene expression in breast cancer biopsies.

We analysed whether the oppositely expressed transcripts encoding IFN-I-related genes correlated with prognosis monitored as relapse-free survival and overall survival. For this, we used data from aggressive triple negative breast cancer (TNBC) and HER2 enriched breast cancer patients, which were compared with the ER positive patient group [44–47]. TNBC and HER2 enriched subtypes are both characterized by the lack of estrogen and progesterone receptor expression, although only HER2 enriched subtype express human epidermal growth factor 2 (HER2). In addition, TNBC and HER2 enriched are more aggressive subtypes compared to the ER positive [44, 48–51].

**Fig. 6:**
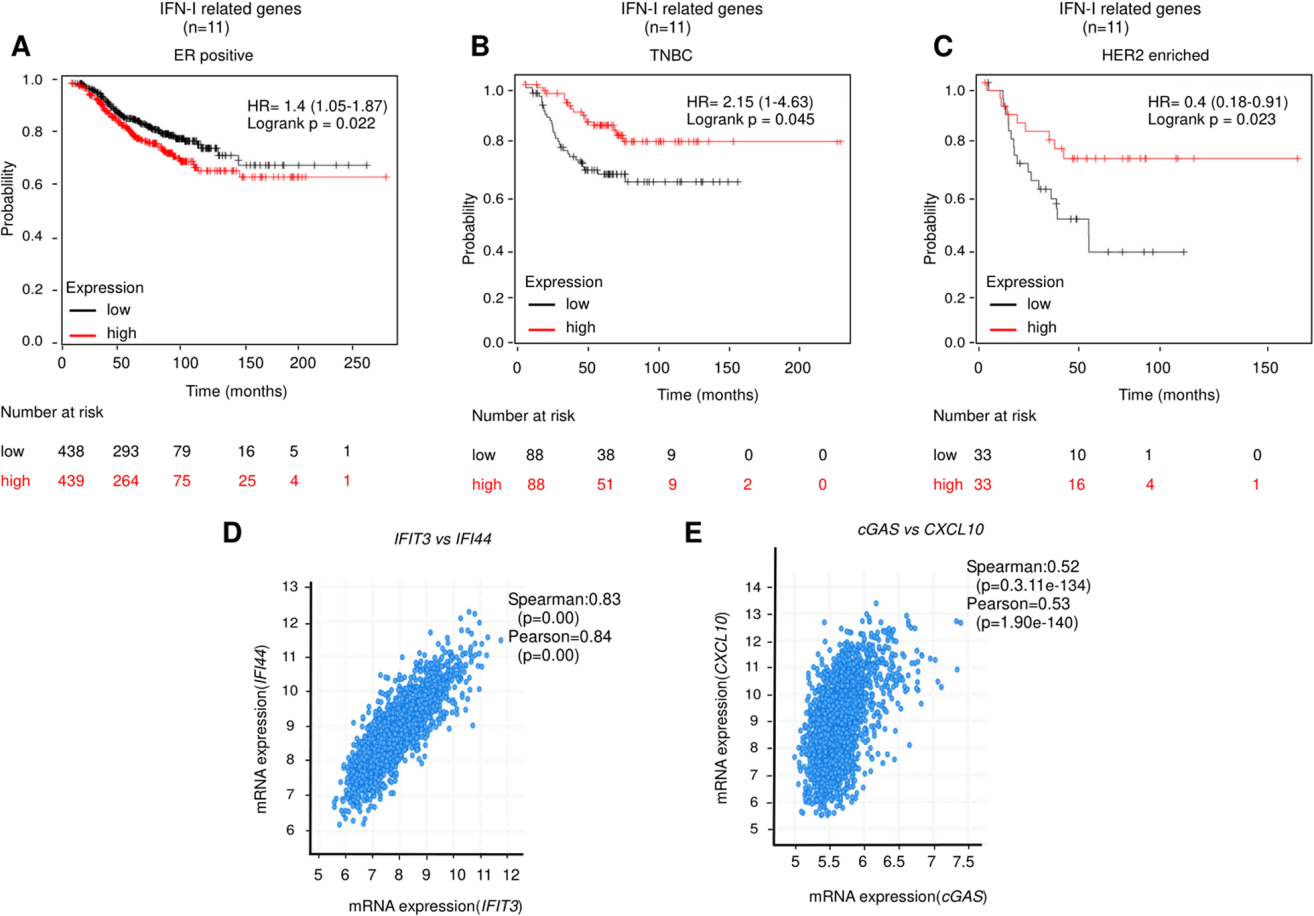
Low IFN-I expression correlates with poor relapse-free survival in breast cancer patients. **A**–**C** Analysis of relationships between gene expression and relapse free survival (RFS) in breast cancer patients using the online tool KM plotter. High and low expression were defined as above and below median. Relationship between mean expression of IFN-I related genes (n = 11) in ER positive (**A**), TNBC (**B**) and HER2 enriched subtypes (**C**). HR, hazard ratio. **D** Dot plot showing a positive correlation between mRNA expression of *IFIT3* and *IFI44* in METABRIC cohort (N = 1904). **E** Dot plot showing a positive correlation between mRNA expression of *cGAS* and *CXCL10* in METABRIC cohort

This analysis showed that lower expression of IFN-I related genes correlate with a reduced relapse-free survival in TNBC as well as HER2 positive patients but not in ER positive cancer patients (Fig. 6A–C). In addition, when we evaluated the overall survival, we found that lower levels of IFN-I related genes in tumor biopsies were unfavorable markers exclusively for TNBC patients (Additional file 1: Fig.S8A–C). We extended these results using the public dataset from the “Molecular taxonomy of breast cancer international consortium” (METABRIC) cohort [43], which facilitated analysis of mRNA expression data from 1904 patients. Here, we observed a strong correlation between several IFN-I genes. For instance, *IFIT3* correlated with *IFI44 *(Fig. 6D), *CXCL10*, *STAT1*, *IFIT1*, *IFI44L*, *ISG15*, *IRF9* and several cytotoxic T cell markers including *CD8A*, *CD8B*, *CD28* (Additional file 2: Table S1). In addition, *cGA*S expression correlated significantly with the expression of several IFN-I induced genes (Fig. 6E and Additional file 3: Table S2). Together these results suggest that lower levels of IFN-I related genes in more aggressive cancer subtypes are associated with unfavourable prognosis.

## Discussion

Although all cancer cells can form a tumor, metastatic cancer cells must endure unique adaptations that help them to undergo immune escape and spread to distant sites [52]. To identify these unique adaptations, we compared metastatic and non-metastatic breast cancer cells when grown in culture, and in the tumors these cells induce when injected in immune-competent mice. We found that the IFN-I response was oppositely regulated in metastatic and non-metastatic cells versus their corresponding tumors. While the IFN-I response was active in the metastatic cell lines when grown in culture, it was markedly dampened in the primary tumor. Interestingly, we also found that the IFN-I response was active in human breast cancer cells in culture, which is associated with an invasive phenotype. Further, the IFN-I response was not active in the non-metastatic cell lines, yet it was induced in the primary tumors. This adaptation may be fundamental to avoid the anti-tumor immune response and metastasis. It is well appreciated that dampened IFN-I signaling aids tumor progression [53]. IFN response can be activated in both cancer cells and immune cells inside the tumor; however, the function is different [54]. In cancer cells, the IFN-I response acts as an alarm system, alerting the immune system to kill the transformed cancer cells. In immune cells, IFN-I response can act as an effector system that contributes to eliminating cancer cells via T cell priming and effector cytokine production [54, 55].The selection of cancer cells in a growing tumor likely involves acquirement of mechanisms that downregulate anti-tumor immunity, including IFN signaling but how cancer cells turn off IFN-I response in the tumors remains incompletely understood. In any case, these findings further underscore potential therapeutic strategies involving reactivation of the IFN-I response to trigger anti-tumor immune reactions. The IFN-I response has long been a key contributor to effective antiviral responses. The induction of IFN-I signaling is essential for the immune system to eliminate cells infected with viruses and intracellular bacterial infections. During infection, the presence of foreign DNA in the cytosol leads to activation of cGAS and subsequent induction of the IFN-I response [53, 55, 56]. Here, we identified elevated levels of cytosolic DNA from micronuclei and mitochondria in metastatic cancer cells in sterile culture conditions. In murine metastatic and invasive human breast cancer cell lines, cytoplasmic DNA foci were often associated with cGAS. This suggests that micronuclei are ruptured and sensed by cGAS. Our data is supported by previous reports showing that disruption of the micronuclear envelope exposes self-DNA to the cytosol, followed by recruitment of cGAS and activation of cGAS-STING signaling [38, 57]. Our observation of mtDNA leakage is supported by our previous findings that show dysfunctional mitochondrial in metastatic cancer cells, characterized by high mtROS production, hyperpolarized mitochondria with higher proton leak, and lower respiratory capacity [58]. We therefore speculate that elevated levels of mitochondrial components and hyperpolarized mitochondria could be a compensatory mechanism to increase ATP production, but also result in mitochondrial damage and mtDNA release in the cytosol. Here we show that the IFN-I response is constitutively active in breast cancer cells with metastatic ability grown in culture, while an effective dampening of this response occurs when they grow as tumors. Likely, this downregulation is either due to stimulated removal of DNA from the cytosol or downregulation of the cGAS-STING signaling pathway in the cancer cells within the tumor. While IFN-I inducers are well-known, negative regulation of IFN-I signaling is poorly understood [59]. Nevertheless, it is well established that cytosolic DNA is degraded by autophagy [60–63]. Even if dampening of the IFN-I response involves elevated autophagy in the cancer cells of a tumor, it remains unknown which factors of the TME cause this effect. It is tempting to speculate that local nutrient restriction could stimulate autophagy in cancer cells of solid tumors. However, the ability to accurately quantify autophagic flux in biopsies is currently limited.

In human tumor biopsies, elevated IFN-I signaling correlates with induced T cell responses against tumor specific antigens [64, 65] and it may represent a mechanism that limits tumor development. In line with this, our METABRIC analysis of 1904 breast cancer patients showed that the expression of several interferon-induced genes correlates with several T cell markers.

Using KM plotter, we observed that reduced expression of the 11 oppositely expressed IFN-related genes predict poor prognosis in both TNBC and HER2 enriched aggressive breast cancer subtypes but not in ER positive subtypes. In the TME, IFN signaling is commonly induced by tumor-associated antigens or due to immunogenic cell death in response to chemotherapy and radiotherapy [66]. In TNBC and HER2-enriched breast cancers patients that undergo trastuzumab monotherapy or in combination with chemotherapy, high tumor-infiltrating lymphocytes were associated with a better prognosis in patients. In contrast, no association was observed in ER-positive patients [67–70]. This could be because CD8^+^ T cells can produce and respond to IFNs mediating antitumor responses [71]. While systemic IFN-I based therapies have been shown to increase the efficacy of the checkpoint inhibitors in TNBC, no predictive values were obtained in the ER-positive [68, 72] patients. This leads to an open question of whether inducing IFN-based immune therapy is beneficial to ER positive patients, which still needs to be addressed. Here, our results highlight the clinical significance of an elevated IFN-I response, supporting the therapeutic potential of increasing IFN-I response in patients where these responses are suppressed. One limitation of this type of data analysis, is the lack of evidence about which cells, immune or non-immune, contributes to the IFN-I response in the patient biopsies. Likely, reduction of IFN response could be either due to the downregulation of immune components that induce IFN-I signaling or due to the stimulated removal of the endogenous DAMPs in the cancer cells in the tumor as mentioned earlier in the discussion [73–75].

Currently, more than 370 (recruiting or active) clinical trials aiming to target IFN-I signaling in cancer patients are ongoing (www.clinicaltrials.gov). However, IFN-associated toxicity has been a significant obstacle for this strategy to be translated to the clinic. Recently, other approaches to activate IFN-I response have been explored including cGAMP-based nanoparticles. cGAMP, is a second messenger that is synthesized in response to cytosolic double stranded-DNA. These nanoparticles enhance the cytosolic delivery of cGAMP and trigger formation of an immune competant TME with enriched T cell infiltration [76]. Alternatively, tumors that are non-responsive to immune checkpoint inhibitors could be transformed into the immune competent tumors by using STING agonist-mediated T-cell priming and infiltration [12, 77–81]. Since STING agonists can redesign the TME to promote stronger antitumor T cell responses [22, 78], they are good candidates for combination with established immunotherapies. However, despite several completed and ongoing Phase II studies detecting signs of clinical activity for STING agonists, no Phase III studies have been registered yet. Even with the best-characterized STING agonist DMXAA, most of the trials with mono and combination therapy have failed due to low efficacy and toxicity issues [77]. These findings highlight the importance of identifying better therapeutic combinations and improving understanding of the underlaying mechanisms controlling this signaling in a complex tumor.

## Conclusion

In this study, we utilized an immunocompetent mouse model of breast cancer to demonstrate that IFN-I signaling represents an important mechanism supporting tumor progression. Further research is needed to uncover the full repertoire of mechanisms that control this immunological switch and find novel strategies to efficiently target aggressive tumors, reduce the risk of metastasis and improve the survival of breast cancer patients.

## Supplementary Information

**Additional file 1**. Supplementary figures S1 to S8 and supplementary methods.

**Additional file 2**. Correlation between IFN-I induced genes expression.

**Additional file 3**. Correlation between cGAS and IFN-I induced genes expression.

## Data Availability

The mass spectrometry proteomics data have been deposited to the ProteomeXchange Consortium via the PRIDE [24] partner repository with the dataset identifier PXD037288. All other data and materials mentioned in this article can be requested by email.

## Abbreviations

Cell: Cellular
Met: Metabolic
Resp: Response
ncRNA: Noncoding RNA
med: Mediated
sig: Signalling
prod: Production
resp: Response
ext: External
Adapt: Adaptive
imm: Immune
som: Somatic
recomb: Recombination
rec: Receptors
Superfam: Superfamily
Ant: Antigen
proc: Process/processing
pres: Presentation
pep: Peptide
Rib Prot: Ribonucleoprotein
Spli: Splicing
Transesterif: Transesterification
React: Reaction
Ade: Adenosine
Sub: Subunit
Org: Organization
Nucelocytopl: Nucleocytoplasmic
End: Endogenous pathway
Mol: Molecule
Reac: Reactive
Spe: Species
Leuk: Leukocyte
Mig: Migration
inf: Inflammatory
cat: Catabolic
cGAS: Cyclic GMP-AMP synthase
cGAMP: Cyclic guanosine monophosphate–adenosine monophosphate
STING: Stimulator of interferon genes
CD45: Cluster of differentiation 45
IFIT3: Interferon-induced protein with tetratricopeptide repeats 3
IFIT1: Interferon-induced protein with tetratricopeptide repeats 1
ISG15: Interferon-stimulated gene 15
IRF3: Interferon regulatory factor 3
IRF7: Interferon regulatory factor 7
XAF1: XIAP-associated factor 1
IGTP: Interferon gamma induced GTPase
STAT1: Signal transducer and activator of transcription 1
OAS3: 2′-5′-Oligoadenylate synthetase 3
GBP2: Guanylate binding protein 2
CXCL10: C-X-C motif chemokine ligand 10
Trim30d: Tripartite motif-containing 30D
IFF7: Interferon regulatory factor 7
H2-T22: Histocompatibility 2, T region locus 22
GBP3: Guanylate binding protein 3
CCL5: C–C motif chemokine ligand 5
H2-Q4: Histocompatibility 2, Q region locus 4
IFI44: Interferon induced protein 44
COX1: Cytochrome c oxidase subunit 1
COX2: Cytochrome c oxidase subunit II
Dloop1: Displacement loop1
Dloop2: Displacement loop1
TBK1: TANK binding kinase 1
