# Supplementary material for: Opposite and dynamic regulation of the interferon response in metastatic and non-metastatic breast cancer
Source: Cell Commun Signal. 2023 Mar 7;21:50. doi: 10.1186/s12964-023-01062-y (PMC9990226; doi:10.1186/s12964-023-01062-y)
Supplement: Supplementary file 2 — Additional file 1. Supplementary figures S1 to S8 and supplementary methods. [file 12964_2023_1062_MOESM2_ESM.docx]

**Fig. S1: Gene ontology analysis of RNA seq. data from metastatic and non-metastatic cell lines and primary tumors reveals dysregulation of multiple biological processes**


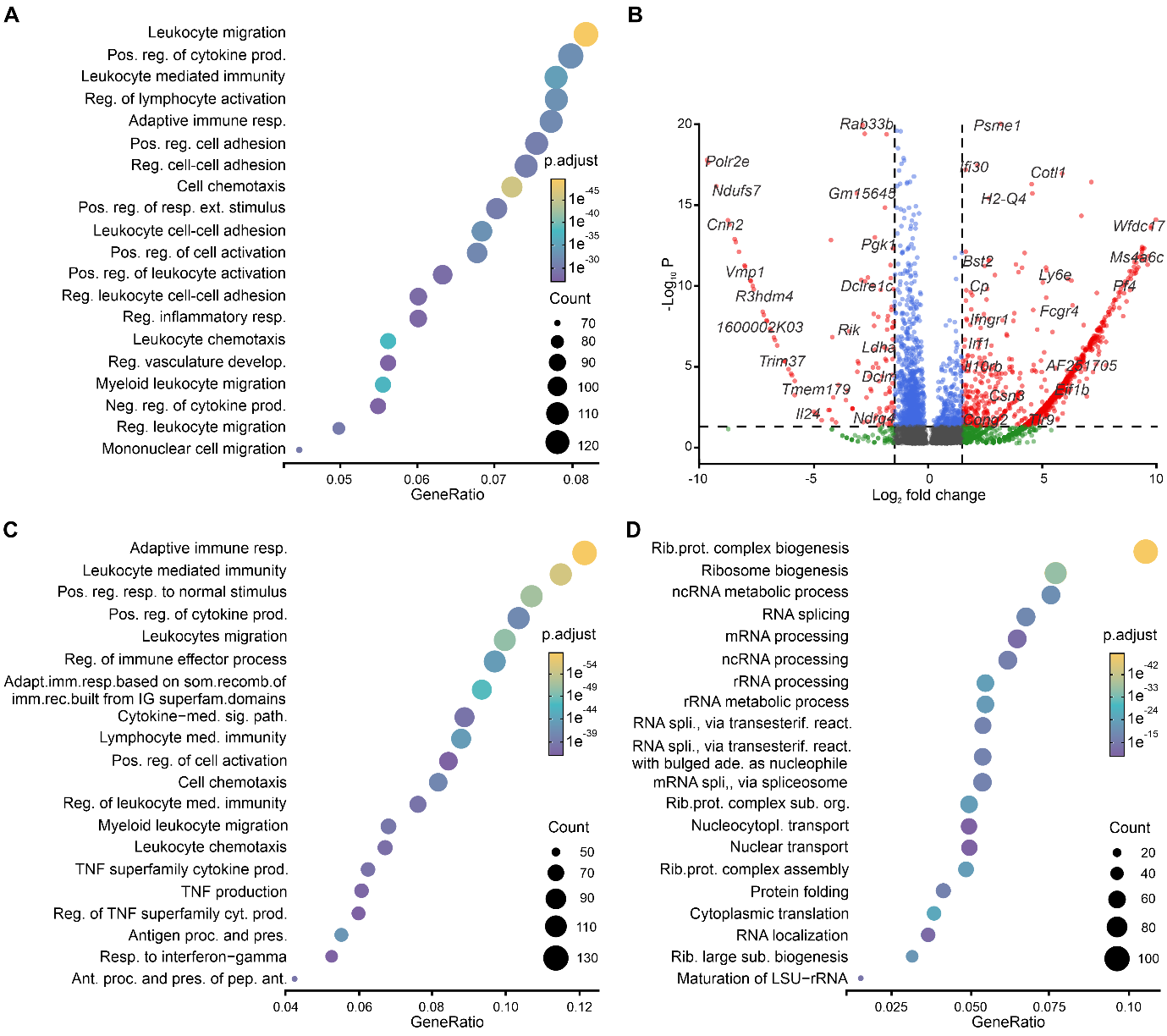


**A)** Gene ontology (GO) analysis for biological processes (BP) of genes with increased expression in 66cl4 tumors versus 66cl4 cell line. **B)** Volcano plots depicting differentially expressed genes from 66cl4 tumors vs 66cl4 cells. Red points represent genes with Log2foldchange(log2FC) within the cut off (±1.5) and adjusted *p*-value <0.05. **C)** GO (BP) functional enrichment analyses of genes highly expressed in 67NR tumor relative to 67NR cells. **D)** Gene ontology analysis for biological processes of genes with low expression in 67NR tumor vs 67NR cells.

**Fig. S2:** **Strategy for comparison of gene expression in metastatic cell and non-metastatic cells in culture and in primary tumors**


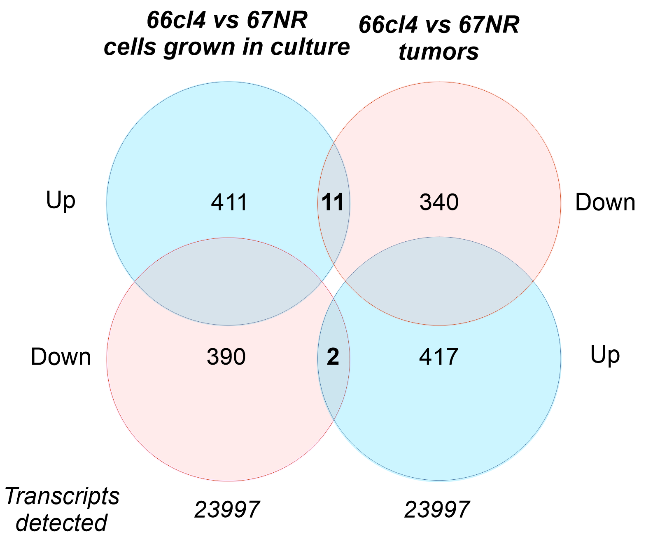


Venn diagram showing differentially expressed genes. Gene expression in the 66cl4 vs 67NR cells in culture and 66cl4 tumors vs 67NR tumors were compared. With a cutoff of log2FC ±1.5 and *p.*adj: <0.05; 411 genes had higher expression and 390 had lower expression in 66cl4 compared to 67NR cells grown inculture. With similar cutoff in the primary tumors formed by 66cl4 and 67NR cells, 340 genes were low expressed, and 417 genes were highly expressed in 66cl4 tumors compared to 67NR tumors. Among the 390 low expressed genes in 66cl4 cells, 2 genes were highly expressed in the 66cl4 tumor. Among 411 genes that were highly expressed in 66cl4 cells, 11 were low expressed in the 66cl4 tumors compared to 67NR tumors. These 11 genes were highly expressed in 66cl4 cells (vs. 67NR cells) while in the 66cl4 tumors they were low expressed (vs 67NR tumors); **“oppositely expressed”**.

**Fig. S3: IFN-I-related gene expression is suppressed in metastatic tumors.**


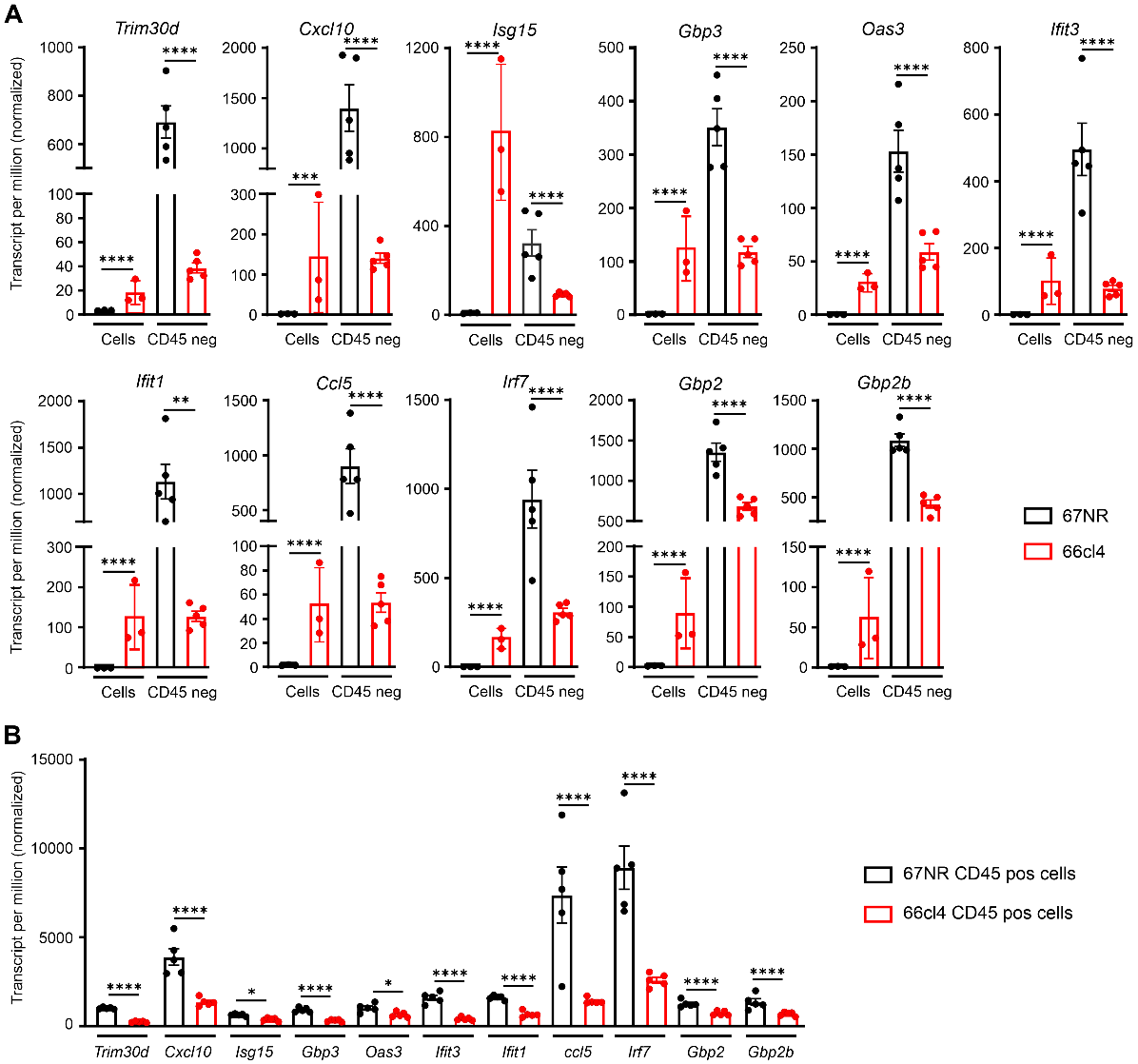


**A**) Significantly upregulated IFN-I genes in 66cl4 cells vs. 67NR cells (N= 3) and downregulated genes in 66cl4 (CD45-negative) vs 67NR (CD45-negative) population (N= 5) sorted from the primary tumors of 66cl4 and 67NR. **B)** Significantly downregulated genes in 66cl4 (CD45-positive) vs 67NR (CD45-positive) population (N= 5) sorted from the primary tumors of 66cl4 and 67NR. Bars represent means ± SEM and each data point represents a single animal (**p* <0.05, ** *p* <0.01, ****p* <0.001 and **** *p* <0.0001). Average expression levels of the selected genes as transcripts per million (TPMs). Bars represent means ± SEM and each data point represents a single animal. Statistical significance was determined using Mann Whitney t-test, **p*< 0.05; ** *p*<0.005; *** *p*<0.0005 vs 67NR tumor.

**Fig. S4: IFN-I proteins are constitutively expressed in metastatic cancer cells in culture but dampened when they form primary tumors**

**A)** Principal Component Analysis of the proteins from 67NR (N= 5) and 66cl4 (N= 6) primary tumor lysates. **B)** Volcano plots depicting differentially expressed proteins in 66cl4 tumor (N= 6) vs 67NR tumor (N= 5). **C)** *Cxcl10* mRNA levels in 67NR and 66cl4 cell lines in culture (N= 3). *Actb* was used as a housekeeping gene and the data was normalized to 67NR. Bars represent means ± SEM (***p* < 0.01, One sample t-test, N= 3). **D)** Total protein stain of western blot membrane shown in Fig.2E. **E)** Total protein stain of western blot membrane shown in Fig. 2F. F) CCL5 and IRF7 immunoblot of protein extracts from 67NR and 66cl4 cell lines (N= 4). G) CCL5 and IRF7 immunoblot of protein extracts from 66cl4 cell line (N= 1) and 66cl4 tumors (N= 5). **H-I)** Total protein stain of western blot membrane shown in F and G respectively.

**Fig. S5: IFN-I expression correlates with the invasive phenotype in human breast cancer cell lines**


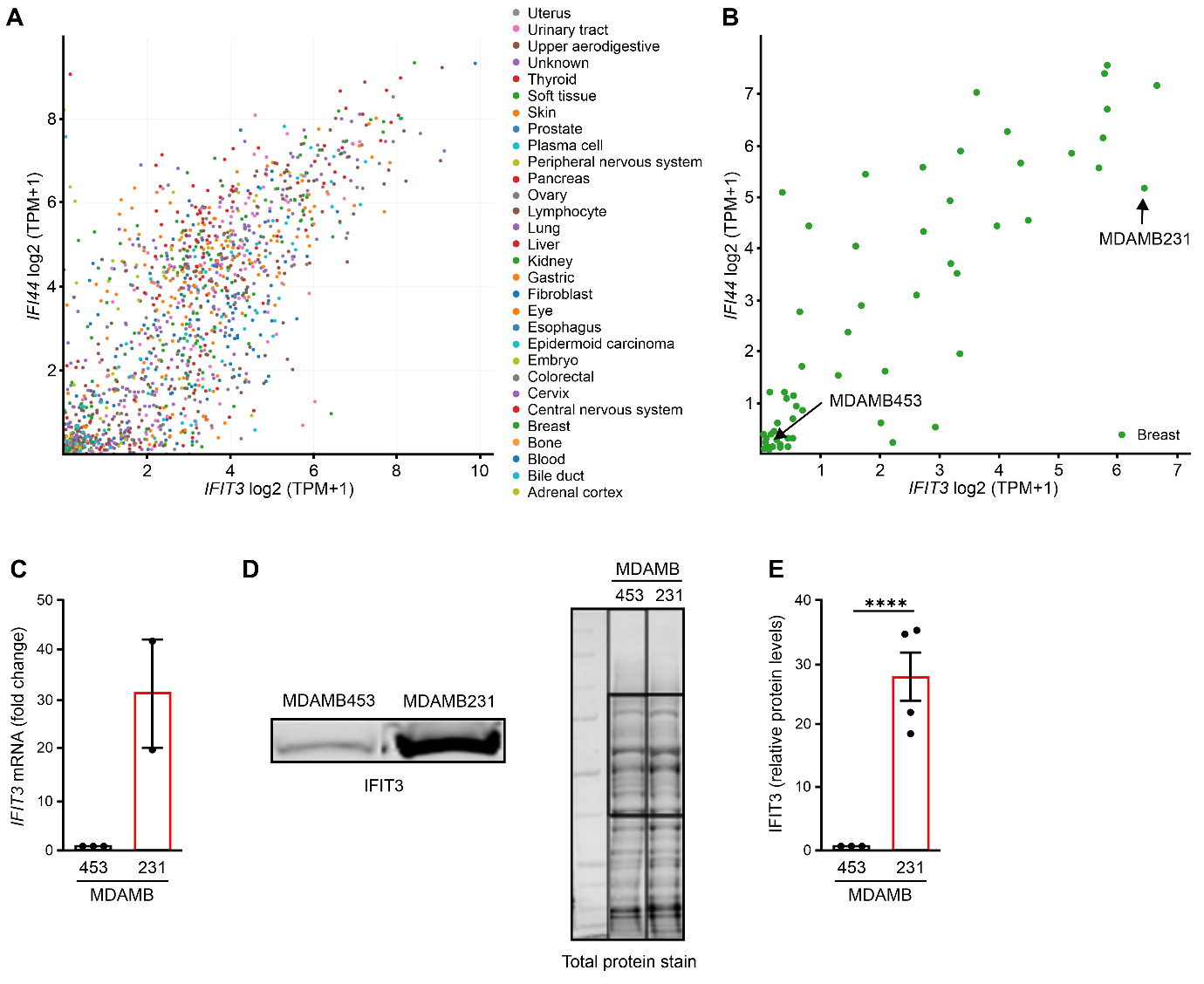


**A)** *IFIT3* and *IFI44* mRNA expression in all cancer cell lines using the Expression 22Q2 Public database in Cancer Cell Line Encyclopedia, CCLE. Each dot represents a cell line and color code is based in their lineage. **B)** *IFIT3* and *IFI44* mRNA expression in breast cancer cell lines. **C)** *IFIT3* mRNA expression levels in MDAMB453 and MDAMB231 cell lines (N= 2). *RNA18SN5* was used as a housekeeping gene, and the data are presented relative to the MDAMB453 cells. **D)** Representative IFIT3 immunoblot of protein extracts of the MDAMB453 and MDAMB231 cell lines (N= 3-4). Total protein staining was used as loading control, and the square indicates the quantified area. **E)** Quantification of IFIT3 protein level. Bars represent mean± SEM relative to MDAMB453 (N= 4, *****p*<0.0001, One sample t-test).

**Fig. S6: Metastatic cancer cells release mtDNA into the cytosol**


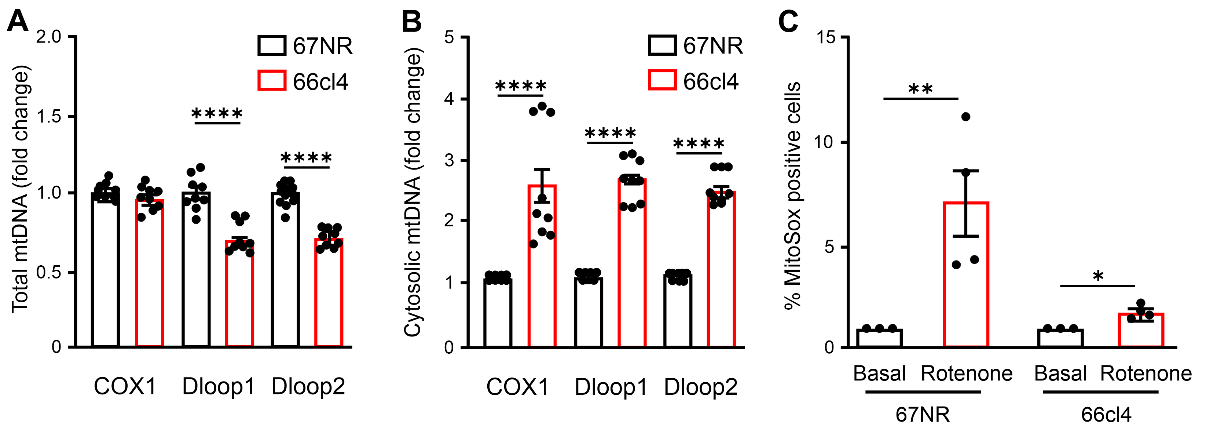


Relative amount of total **(A)** and cytosolic **(B)** mitochondrial DNA (mtDNA) in 67NR and 66cl4 cells, normalized to Tert. Bars represent mean± SEM (N= 3, each in triplicate, t-test, ***p* < 0. 0001, *****p* < 0. 0001). **C)** MitoSOX positive cells (%) in 67NR and 66cl4 cell in presence or absence of rotenone. Bars represent mean± SEM (N= 4, one sample t-test, ***p* < 0. 01, **p* < 0. 05).

**Fig. S7** High levels of STING and pTBK1 are associated with metastatic ability in breast cancer cell lines.


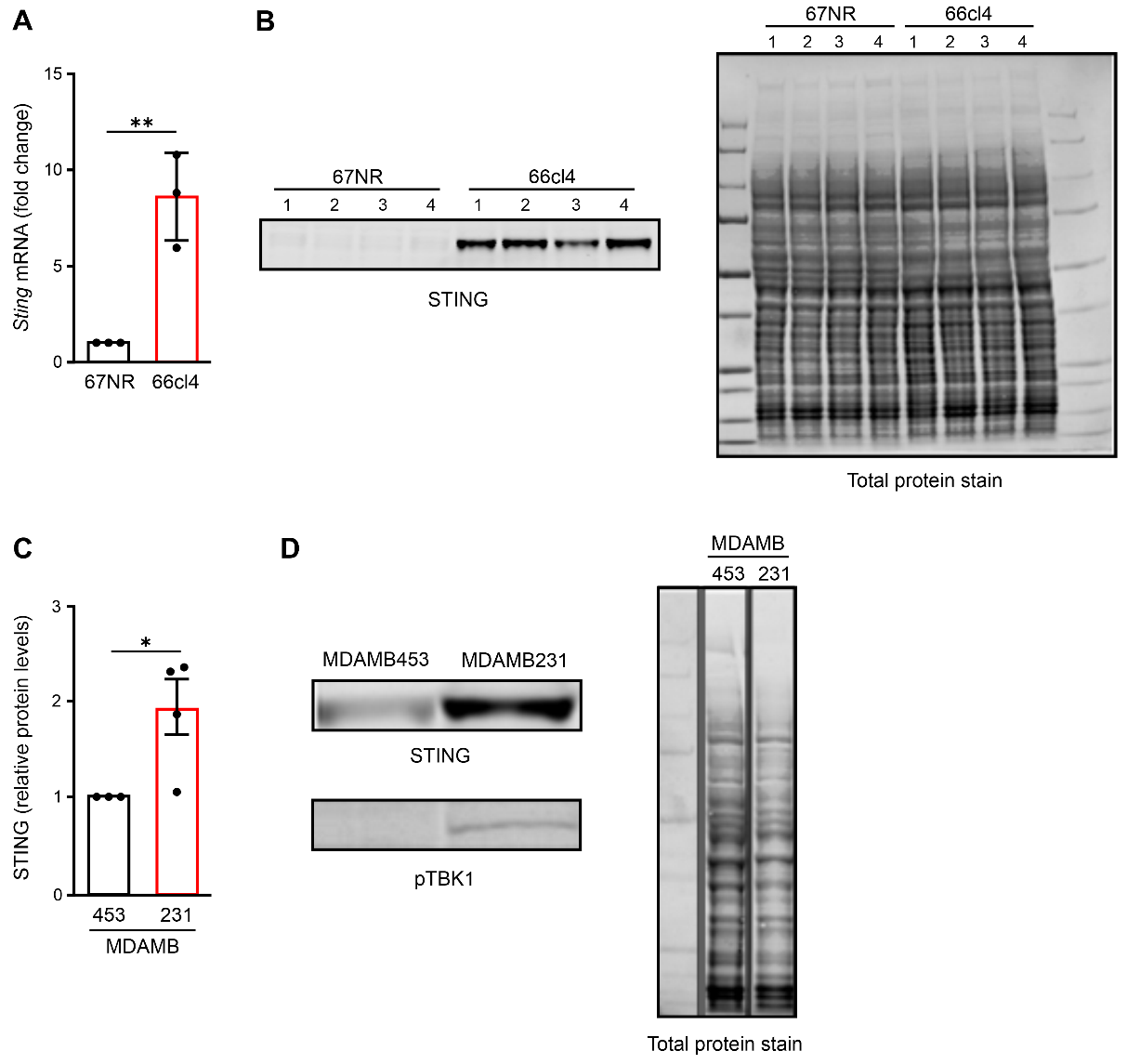


**A)** *Sting* mRNA expression in 67NR and 66cl4 cell lines (N= 3). *Actb* was used for normalization and fold change was calculated relative to 67NR. Bars represent mean ± SEM (N= 3, one sample t-test, ***p* <0.01). **B)** STING immunoblot from 67NR and 66cl4 cell lines lysate. Total protein staining was used as loading control. **C)** STING protein levels in MDAMB453 and MDAMB231 cell lines. Bars represented mean ± SEM relative to total protein staining (N≥ 3, one sample t-test, * *p* <0.05). **D)** Representative STING and pTBK1(Ser172) immunoblot (N= 5) from MDAMB453 and MDAMB231 cell lines. Total protein staining was used as loading control.

**Fig S8: Low IFN-I expression correlates with poor overall survival in breast cancer patients**.


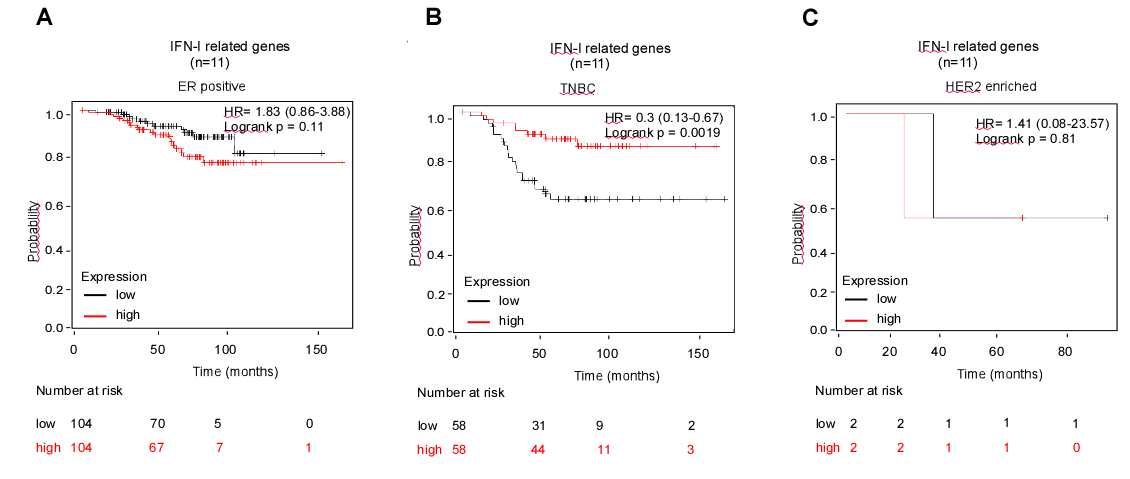


**A-C)** Analysis of relationships between gene expression and overall survival (OS) in breast cancer patients using the online tool KM plotter. High and low expression were defined as above and below median. Relationship between mean expression of IFN-I related genes (n= 11) in ER positive **(A)**, TNBC **(B)** and HER2 enriched **(C)** subtypes. HR, hazard ratio.

**Supplemental Methods**

**Cell lines and Cell culture**

67NR and 66cl4 cells from the 4T1 model were obtained from Barbara Ann Karmanos Cancer Institute, Detroit, MI, USA. In addition to the 4T1 model, two different human breast cancer cell lines that are characterized as non-invasive (MDAMB453) and invasive (MDAMB231) were also used. The MDAMB cell lines both originate from metastases, yet the MDAMB453 is classified as non-invasive based on their Matrix Metallopeptidase 14 (MMP14) expression [1, 2] and is considered as tumorigenic only in semi-solid medium (ATCC). MDAMB453 and MDAMB231 were kindly provided by Dr. Kaisa Lehti. All cell lines were cultured in DMEM (Lonza, BioWhittaker, #BE12- 604F) supplemented with 10% fetal calf serum (Thermo Fischer Scientific,Gibco #10272-106), 2 mM L-Glutamine (Lonza Group, Cat #De-17-605E), and 50 U/ml 50 U/ mL penicillin-streptomycin (Thermo Fischer Scientific, Gibco, #15070-063). Cells were incubated at 37°C with 5% CO2.

**Transcriptome analysis**

As described previously, RNA was isolated from three passages of 67NR and 66cl4 in culture, four and seven primary tumors of 67NR and 66cl4, respectively. Detailed information about sample preparation and data analysis can be found in [3]. The transcriptome data obtained by sequencing mRNA isolated from cells and primary breast tumors of 67NR and 66cl4 is accessible from NCBI (SUB6422687). The data from[3] was reanalyzed into further detail using RStudio version 4.1.2. Genes that had a total count less than 5 were filtered out using filterfun function in RStudio. Differential expression was then determined with DESeq2 using default settings[4]. The presence of differentially expressed genes (DEGs) was determined by comparisons of groups: 66cl4 tumors vs 66cl4 cells ,67NR tumors vs 67NR cells, 66cl4 cells vs 67NR cells and 66cl4 tumor vs 67NR tumor using a significance threshold of *p.adj* < 0.05. The cut off Log2FoldChange of either (±1.5), or (± 1) and *p.*adj < 0.05 was used to define low expressed and high expressed genes. Volcano plots were drawn using the EnhancedVolcano R package (v 1.0.1). Heatmaps of expression data were plotted after log10 transformation and after z scoring the expression values using pheatmap package (v1.0.12). The *hclust* function in pheatmap package was used to performed hierarchical clustering.To evaluate common biological functions of results of DEGs, functional enrichment analyses of all significantly highly expressed genes and all significantly low expressed genes were performed to identify the biological processes involved. The bioconductor package clusterProfiler [5] was used to conduct gene ontology (GO) functional enrichment analyses for biological process (BP), for DEGs from different groups applicable. The plot was visualized using the ggplot2 package (v3.2.1). Analyses for DEGs were performed separately for highly expressed and low expressed genes. For CD45 positive and CD45 negative RNA sequencing, unpublished data was used: GEO NCBI, accession code: GSE211223 (<https://www.ncbi.nlm.nih.gov/geo/info/linking.html>).

**Mice Experiments**

The mice were injected with 1 x 10^6^ viable 66cl4 and 67NR cancer cells into the mammary fat pad. Mice injected with 67NR cells were sacrificed after 3 weeks, while mice injected with 66cl4 were sacrificed after 4 weeks. Tumors were resected and snap frozen in liquid nitrogen and stored at -80^o^C. All experiments involving mice were conducted in accordance with the European Convention for the Protection of Vertebrates used for Scientific Purposes. The mice were housed at the Comparative Medicine Core Facility at NTNU, and the animal studies were approved by the Norwegian Food Safety Authorities (FOTS: 17895 and FOTS: 26021)

**Sample preparation and** **MS analyses**

Small pieces of 67NR and 66cl4 tumors were thawed briefly in lysis buffer and homogenized using 1.4 mm ceramic beads (Precellys, 03961-1-103) in reinforced tubes (KT03961-1-403.2) for 4 cycles á 40 sec homogenization, 2 min break. Lysis buffer: 8 M urea (Merck Millipore, #1084870500) with 0.5 % CHAPS, 100 mM DTT, (Sigma, #646563), 1x Complete® protease inhibitor (Roche, #1187350001) and 2x phosphatase inhibitor cocktail II (Sigma, #P5726) and III (Sigma, #P0044). Homogenized tissue samples were shaken before centrifugation (15 000 g, 20 min, 4°C). Protein concentration was measured at 595 nm using BioRad protein assay dye reagent (Bio-Rad, #500-0006).15µg of each were added to 130µl 100mM ammonium bicarbonate. Proteins were reduced and alkylated with DTT (12mM) for 30min at 55°C. Samples were further alkylated with iodoacetamide (36mM) for 30min at room temperature and dark. Proteins were digested with 250ng trypsin at 37°C overnight and further acidified in acetic acid (0.5%) and desalted using Oasis HLB C^18^ solid phase extraction according to manufacturer’s instructions. After elution of peptides from C^18^, the samples were dried in speedvac and further dissolved in 18 µl 0.1% formic acid and LC-MS/MS were performed on a timsTOF Pro (Bruker Daltonics) connected to a nanoElute (Bruker Daltonics) HPLC. Peptides were separated using a Bruker15 (75µm*15cm) column with running buffers A (0.1% formic acid) and B (0.1% formic acid in acetonitrile) with a gradient from 0% B to 37%B for 100min. The timsTof instrument was operated in the DDA PASEF mode with 10 PASEF scans per acquisition cycle and accumulation and ramp times of 100 millisecond each. The ‘target value’ was set to 20,000 and dynamic exclusion was activated and set to 0.4 min. The quadrupole isolation width was set to 2 Th for m/z < 700 and 3 Th for m/z > 800.

### Proteomics data analysis and bioinformatics analysis

### Proteins were quantified by processing MS data using MaxQuant v.2.0.3.0 [6]. The open workflow provided in FragPipe [7] was used to inspect the raw files to determine optimal search criteria and accordingly search parameters were set as follows: enzyme specified as trypsin with maximum two missed cleavages allowed; deamidation of asparagine/glutamine, oxidation of methionine, and protein N-terminal acetylation as variable modifications; precursor and fragment mass tolerance was set to 20 parts per million (PPM). These were imported in MaxQuant which uses m/z and retention time (RT) values to align each run against each other sample with a minute window match-between-run function and 20 mins overall sliding window using a clustering-based technique. These were further queried against the mouse proteome including isoforms downloaded from Uniprot [8] in 2021 along with MaxQuant’s internal contaminants database using Andromeda built into MaxQuant. Both protein and peptide identifications false discovery rate (FDR) was set to 1%, only unique peptides with high confidence were used for final protein group identification. Peak abundances were extracted by integrating the area under the peak curve. Each protein group abundance was normalized by the total abundance of all identified peptides for each run and protein by calculated median summing all unique and razor peptide-ion abundances for each protein using label-free quantification (LFQ) algorithm [9]  with minimum peptides ≥ 1. LFQ values for all samples were log-transformed with base 2. A correlation heatmap using R package pheatmap [10] was created using these transformed LFQ values and an outlier was removed. The rest of the values representing each condition were subjected to two-sided Student’s t-Tests [11] as implemented in R[12] in order to check the consistency of change. The amount of change was estimated by subtracting the median of these values representing each group (log2 median change). Directionality of the change is encoded within the sign of log2 median change whereby a negative sign reflecting decreased and a positive sign reflecting the increased expression of the respective protein group. Further, to estimate the false-discovery rate (FDR), the T-test p-values were corrected using the Benjamini-Hochberg procedure [13]. Differentially expressed (DE) protein groups were identified at FDR<0.1 and absolute log2 median change >1.5. The DE quantified only in one group were checked if their coefficient-of-variation of log2medianchange was within 5%. The Uniprot accession IDs of these DE were mapped to a volcano-plot using R package ggplot2 [14] . Volcano plots represented in the figures were drawn using the EnhancedVolcano R package (v 1.0.1), and the cut off was set to log2 median change ±1.5 and the corrected T-test p-value of < 0.05. log2 median change is represented as log2FoldChange throughout the paper.Functional enrichment analyses of all differentially expressed proteins were performed similar as for the transcriptome data.

**Quantitative real-time PCR**

cDNA was synthesized from 500 ng total RNA by High-Capacity cDNA Reverse Transcription Kit (Invitrogen, #4368814). Quantitative real-time PCR (RT-PCR) was performed in 20 µl reactions containing 10 µl of 2X QuantiTect SYBR Green PCR master mix (Qiagen), 2 µl 10X QuantiTect Primer Assay and 8 µl of the sample containing 4 ng of cDNA. Primers are specified under Primer section. RT-PCR was performed on the StepOne plus system (Applied Biosystems) using the following cycling conditions: 95ºC for 15 min, 40 cycles of 94°C for 15 sec, 55°C for 30 sec and 72°C for 30 sec. Relative gene expression levels were calculated with the 2^(-ΔΔCT) method. Transcripts were normalized to *Actb* for mouse and *RNA18SN5* for human cell lines.

**Primers**

QuantiTect Primer Assays were purchased from Qiagen: Mm_*Actb*_2_SG (QT01136772), Hs_*IFIT3*_1_SG (QT00100030), Mm_*Cxcl10*_1_SG (QT00093436), Mm_*Tmem173*_2_SG (QT01045618, for *Sting*). The following primers were purchased from Merck: mDloop1 Fw (AATCTACCATCCTCCGTGAAACC), Rev (TCAGTTTAGCTACCCCCAAGTTTAA); mDloop2 Fw (CCCTTCCCCATTTGGTCT), Rev (TGGTTTCACGGAGGATGG); mCOX1 Fw (GCCCCAGATATAGCATTCCC), Rev (GCCCCAGATATAGCATTCCC); m18SrRNA Fw (TAGAGGGACAAGTGGCGTTC), Rev (CGCTGAGCCAGTCAGTGT); mTert Fw (CTAGCTCATGTGTCAAGACCCTCTT), Rev (GCCAGCACGTTTCTCTCGTT); h18SrRNA Fw (GTAACCCGTTGAACCCCATT), Rev (CCATCCAATCGGTAGTAGCG).

**Immunoblotting**

Protein concentration was measured at 595 nm using BioRad protein assay dye reagent (Bio-Rad, #500-0006). Equal amounts of proteins (50µg) were run on Invitrogen NuPAGE Bis-Tris protein gels, transferred onto nitrocellulose membranes using iBlot dry blotting system, blocked in Intercept (TBS) blocking buffer (Li-Cor, mixed 1:1 with TBS containing 0.1% Tween 20 (TBST), and probed with antibodies as listed under antibodies section. Membranes were scanned and analyzed using an Odyssey CLx Infrared Imaging System and Image Studio v3.1 and v5.2 (Li-Cor). For normalization we used either Revert 700 total protein stain (Li-Cor) or antibody against ERK1/2.

**Antibodies**

The following antibodies were diluted in Intercept blocking buffer/TBST-mix 1:1 and used for immunoblotting: CXCL10 (Abcam, #ab9938, 1:1000), CCl5/RANTES (E9S2K) (Cell Signaling Technology (CST), #36467,1:1000). IRF7 (CST), #72073,1:1000), pIRF3 (CST, #29047,1:1000), STING/TMEM173 (D2P2F) (CST, #13647, 1:1000), pTBK1-Ser172 (CST, #D52C2, 1:1000), ERK1/2 (CST, #9107S, 1:2000) or IFIT3 (E-10) (Santa Cruz Biotechnology #sc-393396,1:500). Proteins of interest were detected with near-infrared fluorescent secondary antibodies (Li-Cor; IRDye 800CW and IRDye 680CW diluted 1:10 000 and 1:20 000, respectively, in Intercept blocking buffer/TBS-mix 1:1 (no Tween)).

**Immunofluorescence**

Cells were grown on high precision cover glass (thickness 0.17 ± 0.005mm; Marienfeld) until desired confluency, then fixed with ice cold methanol (10-15 min at -20 ºC) and permeabilized with 0.05% saponin in PBS for 5 min at room temperature (RT). Cells were then stained with the following antibodies to detect cGAS and Lamin A: rabbit anti-human cGAS (D1D3G) (Cell Signaling #15102; 1:100), rabbit anti-mouse cGAS (D3080) (Cell Signaling #31659; 1:500), mouse anti-Lamin A (abcam #ab8980;1:100), donkey anti-mouse Alexa Fluor 568 (Molecular Probes #A10037; 1:500), and donkey anti-rabbit Alexa Fluor 488 (Jackson #711605152; 1:500). Primary and secondary antibodies were diluted in PBS containing 0.05% saponin and incubated for 1–2 h at RT for primary and 30min-1h for secondary antibodies. After antibody staining, the samples were mounted on microscope slides (Menzel–Glaser) with Mowiol (Sigma Aldrich #81381) containing 10 µg/ml Hoechst 33342 (Invitrogen #H3570) and kept in the dark and in a cold room until imaged. For quantifications, cells were imaged on a Nikon ECLIPSE Ti2-E inverted microscope (Nikon Corp, Tokyo, Japan) equipped with a CSU-W1 dual spinning disc (50 µm pinholes & 50 µm pinholes with microlenses) confocal unit (Yokogawa Electric Corp, Tokyo, Japan), a Prime BSI sCMOS camera (Teledyne Photometrics, Tucson, AZ, US), a laser unit with 405/488/561/638nm lasers (120/100/100/100mW), and BrightLine single-band bandpass filters (447/60nm, 525/50nm, 600/52nm, 708/75nm). ROI were randomly selected and Z-stacks with sectioning of 0.6 µm were collected with a 40X Plan Apo λ objective (NA 0,95, Air). The total number of cells was assessed by segmentation of nuclei using NIS-Elements AR (Nikon) and then the fraction of micronucleated cells and cGAS positive micronuclei were scored. More than1000 cells were counted for each experiment. Representative images were taken at Zeiss LSM 780 confocal microscope a laser diode 405–430 CW (405 nm), a DPSS-561 10 (561 nm), and (Argon laser (488 nm). The objective used was a Zeiss plan-apochromat ×63/1.40 oil DIC M27.

**Analysis of mitochondrial membrane potential:**

The cells were cultured until 80% confluency, and then stained with 200nM of tetramethylrhodamine, ethyl ester, perchlorate (TMRE, Invitrogen) and 300nM Mitotracker Green (MTG, Invitrogen) for 30 minutes at 37°C. The cells were harvested by trypsinization, and then stained with Fixable viability stain 780 (FVD, Invitrogen, 1:1000 in PBS) for 30 min on ice. The cells were washed twice and resuspended in FACS buffer (PBS with 2% FCS and 0.2 mM EDTA) and run on a BD LSR II flow cytometer in biological and technical triplicates recording 50,000 events per well. The fcs files were analyzed in FlowJo10.2 software. Cell gates were set after the exclusion of duplets and dead cells, followed by gating on TMRE and MTG positive cells. Mitochondrial abundance and mitochondrial membrane potential (MMP) was measured with median fluorescence intensity (MFI) of MTG and TMRE, respectively. The MFI of TMRE was normalized to MFI of MTG to determine the mitochondrial activity of the total mitochondria present within each cell population.

**Detection of mitochondrial reactive oxygen species**

The cells were cultured until 80% confluency, and then stained with 5µM of MitoSOX™ Red mitochondrial superoxide indicator (Invitrogen) for 1 hour at 37°C. The cells were harvested by trypsinization, washed twice in PBS and stained with Fixable viability stain 780 (FVD, Invitrogen, 1:1000) for 30 mins on ice. The cells were washed twice and resuspended in FACS buffer (PBS with 2% FCS and 0.2 mM EDTA) and run on a BD LSR II flow cytometer in biological quadruplicates and technical triplicates recording 50,000 events per well. The fcs files were anlayzed in FlowJo 10.2 software. Cell gates were set after the exclusion of duplets and dead cells, followed by gating on MitoSOX positive cells. The percentage of cells positive for MitoSOX was used to determine the mitochondrial ROS within each cell population.

**Detection of total and cytosolic mtDNA**

The cells were cultured in 6-well plates until 80% confluency was achieved. For isolation of cytosolic DNA, cells were washed once in PBS, scraped and lysed on ice using 100 µl 0.1% NP-40, transferred to eppendorf tubes and incubated for 15-20 min on ice, and centrifuged at 16,000 × g for 20 min at 4°C [15]. The supernatants were transferred to new tubes and stored at -80^o^C until isolation of DNA. For isolation of total cellular DNA, the cells were harvested by trypsinization, centrifuged at 340 × g for 5 min, the supernatants were removed and the pellets were stored at -80^o^C until isolation of total (nuclear, mitochondrial and cytosolic) DNA. All DNA samples were isolated by QIAamp DNA mini kit (#51306). To avoid clogging of the columns, we used only half of the sample material for the isolation procedures. For isolation of total DNA, the cell pellets were dissolved in 185 µl of PBS, the exact final volume was determined, half of this was removed and kept as backup while the remaining was adjusted up to 200 µl with PBS. The further protocol including proteinase K (20 µl), AL-lysis buffer (200 µl) and ethanol (200 µl) was as described by the manufacturer, and the samples were eluted with 200 µl water. For isolation of cytosolic DNA, the exact sample volumes were determined, half was removed and kept as backup while the remaining was adjusted up to 100 µl with 1% NP-40 lysis buffer. To get samples as comparable as possible to the samples used for isolation of total DNA, we added PBS (200 µl), AL-lysis buffer (100 µl) and ethanol (200 µl) before proceeding as described by the manufacturer and eluting in 200 µl water. Quantitative PCR was performed on equal volumes of all DNA samples from both cell lines using primers specific for mitochondrial DNA (COX1, Dloop1, Dloop2) and nuclear DNA (18S rRNA and Tert) (primer sequences above under primer section). Using this extraction protocol, nuclear DNA was present only in very low amounts in the cytosolic fraction (CT-values for 18S rRNA ranging from 29.8 – 31.4 and Tert from 29.8 to undetermined), indicating that nuclear rupture occurred at minimum. The relative abundance of both total and cytosolic mtDNA was calculated relative to the non-metastatic cell line 67NR (see explanation below).

Calculations used on qPCR data to determine content of mitochondrial DNA in total and cytosolic DNA

The following calculations were done, based on [16] with small adjustments:

For total DNA 🡪

ΔCT_tot_ = CT (mitochondrial primer) – CT (nuclear primer = 18S rRNA or Tert) (to relate the amount of mitochondrial DNA in the total DNA to cell numbers)

Average ΔCT_tot_ = average of ΔCT_tot_ values obtained for the total DNA from your sample (67NR and 66cl4)

ΔΔCT_tot_ = ΔCT_tot_ (for your sample of interest; 67NR or 66cl4) - average ΔCT_tot_ 67NR (to evaluate if there is a difference in mitochondrial DNA per cell in total DNA from 66cl4 as compared to 67NR)

Relative abundance of mitochondrial DNA in the total DNA sample = 2^^(-^^ΔΔCTtot)^

For cytosolic DNA 🡪

Here the average ΔCT_tot_ for each cell type (see above) is included in the calculations.

ΔCT_cyt_ = CT (mitochondrial primer; 67NR or 66cl4) – average ΔCT_tot_ (for 67NR or 66cl4) (the amount of mitochondrial DNA in cytosolic DNA from each cell line is related to the total amount of mitochondrial DNA per cell for that particular cell line)

Average ΔCT_cyt_ = average of ΔCT_cyt_ values obtained for the reference sample = 67NR

ΔΔCT_cyt_ = ΔCT (for your sample of interest; cyt DNA 67NR or 66cl4) – average ΔCT_cyt_ 67NR (to evaluate if there is a difference in mitochondrial DNA per cell in cytosolic DNA from 66cl4 as compared to 67NR)

Relative abundance of mitochondrial DNA in the cytosolic DNA sample = 2^ ^(-^^ΔΔCTcyt)^

**Use of public databases**

Kaplan-Meier plotter [17] is an online database that utilizes data from multiple cDNA microarrays for examining prognostic markers in several cancer types, including breast cancer [18, 19]. Relapse-free survival (RFS) and overall survival (OS) of IFN-I markers were analyzed in estrogen receptor (ER) positive (ER positive subtype), ER-negative, progesterone receptor (PR)-negative and human epidermal growth factor receptor 2 (HER2) negative (Triple negative breast cancer (TNBC) subtype and HER2 enriched subtype (ER negative, PR negative, Her2 positive) [20]. A set off 11 IFN-I genes were analyzed using the mean expression of 11 different trancripts. Broad Institute Cancer Cell Line Encyclopedia (CCLE), (<https://portals.broadinstitute.org/ccle>) [21] was used for gene expression analysis in human breast cancer cell lines.cBioPortal [22] is an open access database that allows visualization and analysis of large-scale cancer genomics data sets[23, 24].Gene exepression (mRNA expression) data were used to identify the correlation between expression of various genes within the Molecular taxonomy of breast cancer international consortium (METABRIC) cohort [25-28].

**REFERENCES**

1. von Nandelstadh, P., et al., *Actin-associated protein palladin promotes tumor cell invasion by linking extracellular matrix degradation to cell cytoskeleton.* Mol Biol Cell, 2014. **25**(17): p. 2556-70.

2. Sugiyama, N., et al., *EphA2 cleavage by MT1-MMP triggers single cancer cell invasion via homotypic cell repulsion.* J Cell Biol, 2013. **201**(3): p. 467-84.

3. Neckmann, U., et al., *GREM1 is associated with metastasis and predicts poor prognosis in ER-negative breast cancer patients.* Cell Commun Signal, 2019. **17**(1): p. 140.

4. Love, M.I., W. Huber, and S. Anders, *Moderated estimation of fold change and dispersion for RNA-seq data with DESeq2.* Genome Biol, 2014. **15**(12): p. 550.

5. Yu, G., et al., *clusterProfiler: an R package for comparing biological themes among gene clusters.* OMICS, 2012. **16**(5): p. 284-7.

6. Tyanova, S., T. Temu, and J. Cox, *The MaxQuant computational platform for mass spectrometry-based shotgun proteomics.* Nat Protoc, 2016. **11**(12): p. 2301-2319.

7. Geiszler, D.J., et al., *PTM-Shepherd: Analysis and Summarization of Post-Translational and Chemical Modifications From Open Search Results.* Mol Cell Proteomics, 2021. **20**: p. 100018.

8. UniProt. *UniProtKB - H3BJL3 (H3BJL3_MOUSE)*. 2021 October 2021]; Available from: (<https://www.uniprot.org/proteomes/UP000000589>.

9. Cox, J., et al., *Accurate proteome-wide label-free quantification by delayed normalization and maximal peptide ratio extraction, termed MaxLFQ.* Mol Cell Proteomics, 2014. **13**(9): p. 2513-26.

10. *pheatmap: Pretty Heatmaps*. Available from: <https://cran.r-project.org/web/packages/pheatmap/index.html>.

11. Student, *The Probable Error of a Mean.* Biometrika, 1908. **6**(1): p. 1-25.

12. project, R. *The R Project for Statistical Computing*. Available from: <https://www.r-project.org/>.

13. Hochberg, Y.B.a.Y. *Controlling the False Discovery Rate: A Practical and Powerful Approach to Multiple Testing*. 1995; Available from: <https://www.jstor.org/stable/2346101>.

14. Wickham, H. *ggplot2: Elegant Graphics for Data Analysis*. 2009; Available from: <https://www.springer.com/gp/book/9780387981413>.

15. Bronner, D.N. and M.X. O'Riordan, *Measurement of Mitochondrial DNA Release in Response to ER Stress.* Bio Protoc, 2016. **6**(12).

16. Bryant, J.D., et al., *Assessing Mitochondrial DNA Release into the Cytosol and Subsequent Activation of Innate Immune-related Pathways in Mammalian Cells.* Curr Protoc, 2022. **2**(2): p. e372.

17. kmplot.com. 2019; Available from: <https://kmplot.com/analysis/index.php?p=service&cancer=breast>.

18. Lanczky, A., et al., *miRpower: a web-tool to validate survival-associated miRNAs utilizing expression data from 2178 breast cancer patients.* Breast Cancer Res Treat, 2016. **160**(3): p. 439-446.

19. Gyorffy, B., et al., *An online survival analysis tool to rapidly assess the effect of 22,277 genes on breast cancer prognosis using microarray data of 1,809 patients.* Breast Cancer Res Treat, 2010. **123**(3): p. 725-31.

20. Plotter, K.-M. *Kaplan-Meier Plotter*. 2019 [cited 2019 June 2019]; Available from: <https://kmplot.com/analysis/index.php?p=service>.

21. Ghandi, M., et al., *Next-generation characterization of the Cancer Cell Line Encyclopedia.* Nature, 2019. **569**(7757): p. 503-508.

22. cBioPortal. *cBioPortal.org*. 2019; Available from: <http://www.cbioportal.org>.

23. Cerami, E., et al., *The cBio cancer genomics portal: an open platform for exploring multidimensional cancer genomics data.* Cancer Discov, 2012. **2**(5): p. 401-4.

24. Gao, J., et al., *Integrative analysis of complex cancer genomics and clinical profiles using the cBioPortal.* Sci Signal, 2013. **6**(269): p. pl1.

25. Curtis, C., et al., *The genomic and transcriptomic architecture of 2,000 breast tumours reveals novel subgroups.* Nature, 2012. **486**(7403): p. 346-52.

26. Pereira, B., et al., *The somatic mutation profiles of 2,433 breast cancers refines their genomic and transcriptomic landscapes.* Nat Commun, 2016. **7**: p. 11479.

27. Rueda, O.M., et al., *Dynamics of breast-cancer relapse reveal late-recurring ER-positive genomic subgroups.* Nature, 2019. **567**(7748): p. 399-404.

28. cbioportal. *Metabric cohort*. 2021; Available from: <https://www.cbioportal.org/results>.
